# Functional Foods as Modulators of Epigenetic Mechanisms Affecting Metabolic Health in Adolescence

**DOI:** 10.3390/ijms27042066

**Published:** 2026-02-23

**Authors:** Natalia Kurhaluk, Renata Kołodziejska, Zbigniew Mazur, Oleksandr Lukash, Oleksandr Yakovenko, Halina Tkaczenko

**Affiliations:** 1 Institute of Biology, Pomeranian University in Słupsk, Arciszewski St. 22B, 76-200 Słupsk, Poland; zbigniewmazur1@wp.pl; 2 Department of Medical Biology and Biochemistry, Collegium Medicum in Bydgoszcz, Nicolaus Copernicus University in Toruń, M. Karłowicz St. 24, 85-092 Bydgoszcz, Poland; renatak@cm.umk.pl; 3 Department of Ecology, Geography and Nature Management, T.H. Shevchenko National University “Chernihiv Colehium”, 53 Hetmana Polubotka St., 14013 Chernihiv, Ukraine; lukash2011@ukr.net (O.L.); ajakov2@gmail.com (O.Y.)

**Keywords:** functional foods, epigenetics, metabolic health, DNA methylation, histone modification, microRNA, polyphenols, omega-3 fatty acids, dietary fibre, obesity, insulin resistance, metabolic programming

## Abstract

Adolescence represents a critical window of metabolic plasticity, during which profound hormonal, neurobiological, and physiological remodelling increases susceptibility to nutritional exposures. In parallel with the rising prevalence of obesity, insulin resistance, metabolic syndrome, and non-alcoholic fatty liver disease among young people, there is growing interest in the potential for functional food components to modulate epigenetic pathways that govern metabolic programming. This narrative review synthesises current evidence (2015–2025) from PubMed, Scopus, Web of Science, and Embase to elucidate how diet-derived bioactive compounds influence epigenetic regulation relevant to adipogenesis, appetite control, insulin signalling, and lipid homeostasis during adolescence. Particular emphasis is placed on molecular mechanisms, including DNA methylation changes in genes regulating adipocyte differentiation, hypothalamic neuropeptide expression, and pancreatic β-cell function; histone modifications, such as acetylation and methylation events that remodel chromatin accessibility in metabolic tissues; and modulation of microRNA networks implicated in lipid metabolism, inflammatory signalling, and insulin secretion. Furthermore, the review examines the interplay between diet, the gut microbiota, and the epigenome, highlighting the role of microbially derived short-chain fatty acids (SCFAs) as endogenous histone deacetylase inhibitors and mediators of epigenetic remodelling in adipose tissue. By linking these mechanisms to specific functional food components, including polyphenols, long-chain omega-3 fatty acids, fermentable dietary fibre, and other bioactive molecules, we demonstrate how nutritional signals can counteract maladaptive metabolic trajectories and potentially reduce the intergenerational transmission of metabolic risk. A deeper understanding of these epigenetic effects provides the foundation for developing personalised nutrition strategies aimed at preventing metabolic disorders from emerging during adolescence and beyond.

## 1. Introduction

Adolescence represents a dynamic and highly plastic developmental period that begins with the onset of puberty and extends into early adulthood. It is typically subdivided into early adolescence (10–14 years), late adolescence (15–19 years), and emerging adulthood (20–24 years), as outlined by Das et al. [1]. During these stages, profound physiological, neurological, and metabolic transformations occur in parallel with rapidly evolving cognitive, social, and behavioural capacities. Collectively, these changes shape the trajectory of human maturation and, as emphasised by Kemp et al. [2], render individuals uniquely sensitive to environmental influences, including dietary exposures. During this period, adolescents progressively gain autonomy from caregivers in various areas, including decision-making, social functioning, and food acquisition, preparation, and consumption. This process has been extensively documented by Neufeld et al. [3] and Meeus [4]. The dietary behaviours of adolescents increasingly reflect a complex interplay of environmental, psychosocial, and cultural factors, such as food availability, peer norms, socioeconomic status, and internalised beliefs. Consequently, the adolescent nutritional landscape becomes highly variable and particularly susceptible to suboptimal choices, as noted by Das et al. [1].

A growing body of evidence indicates that the overall dietary quality of adolescents remains suboptimal. Gu and Tucker [5] demonstrated that only a minority of American adolescents meet the recommended daily intake of fruits and vegetables, a finding corroborated by Moore et al. [6]. Similarly, Kim et al. [7] reported low adherence to the Mediterranean diet among US youth, although slightly higher adherence was observed among Mexican-American adolescents compared with their peers from other ethnic groups. These patterns are not confined to the United States. Longitudinal studies from Western Europe reveal comparable declines in fruit and vegetable consumption, accompanied by an increased intake of sugar-sweetened beverages during adolescence [8]. Breakfast quality also remains inadequate, with only a small proportion of children and adolescents consuming nutrient-dense morning meals [9]. Skipping breakfast is becoming increasingly prevalent among older adolescents [10] and has been associated with poorer overall diet quality, higher body mass index, an elevated risk of metabolic and cardiovascular disturbances, and adverse mental health outcomes [11,12]. Although daily breakfast consumption is relatively common among European children, its frequency declines markedly with advancing age during adolescence [9].

In addition to irregular meal patterns, adolescents exhibit a higher propensity for snacking, skipping meals, eating out, consuming fast food, and dieting to control their weight—behaviours that are particularly prevalent among girls [1]. A high intake of ultra-processed foods (UPFs), which are rich in refined carbohydrates, unhealthy fats, and additives, has been shown to disrupt gut microbiota composition, impair intestinal barrier integrity, and alter gastrointestinal motility. These alterations ultimately promote low-grade inflammation and dysregulation of the gut–brain axis, mechanisms implicated in the pathophysiology of functional gastrointestinal disorders (FGIDs), as described by Calcaterra et al. [13]. Furthermore, undernutrition during early life, whether in childhood or adolescence, has been associated with an increased risk of metabolic syndrome in adulthood [14]. Many adolescents fail to meet the recommended intake of essential vitamins and minerals, with deficiencies more prevalent among girls. Concurrently, excessive consumption of total and saturated fats, cholesterol, sodium, and simple sugars remains common [1].

Nutritional behaviours established during adolescence have long-term consequences for adult health, as demonstrated by Schneider et al. [15] and Buckland et al. [16]. Risk factors for cardiometabolic diseases, including type 2 diabetes and cardiovascular disorders, frequently emerge during this developmental stage and may predict subsequent health trajectories [17,18]. Although dietary quality tends to improve modestly from adolescence to adulthood, the intake of several key nutrients remains suboptimal [19]. As Kemp et al. [2] argue, dietary patterns formed during youth may therefore exert lasting physiological effects.

Recent advances in molecular nutrition have highlighted the capacity of dietary components to modulate metabolic processes by altering chromatin structure and regulating transcriptional and translational activity [20]. Epigenetic mechanisms, including DNA methylation, histone modifications, and microRNA expression, act as critical interfaces linking environmental exposures, genetic predispositions, and metabolic outcomes [21]. Despite these advances, the extent to which functional foods and their bioactive constituents influence epigenetic pathways relevant to metabolic health during adolescence remains insufficiently understood.

Adolescence is characterised by heightened metabolic and epigenetic plasticity; therefore, it is particularly important to define functional foods precisely for this age group. The broad descriptions employed by the Food and Agriculture Organisation, the Mayo Clinic [22], and the Academy of Nutrition and Dietetics [23] risk conflating conventional healthy foods with deliberately engineered products. Such ambiguity may obscure which dietary factors meaningfully influence epigenetic programming during this critical developmental stage [24]. Given the rapid growth, hormonal fluctuations, and remodelling of metabolic pathways that occur during adolescence, distinguishing between naturally nutrient-dense foods and novel formulations enriched with bioactive compounds or live microorganisms is essential. This differentiation is emphasised in Temple’s revised definition, as well as in frameworks proposed by the Institute of Food Technologists [25] and the Functional Food Centre [26,27,28,29]. Establishing such conceptual clarity is crucial for identifying dietary components capable of modulating DNA methylation, histone modifications, and microbiome-mediated signalling.

Functional foods enriched with omega-3 fatty acids, plant sterols, probiotics, prebiotics, or concentrated polyphenols may exert measurable effects on pathways related to inflammation, insulin sensitivity, and long-term metabolic risk—processes that are, in part, regulated through epigenetic mechanisms during adolescence. Emerging clinical evidence suggests that targeted formulations of this kind have the potential to influence cardiometabolic and immune outcomes [30]. These findings underscore the need for a rigorous, mechanistically grounded definition of functional foods when evaluating their role in adolescent health and epigenetic regulation.

To address this gap, the present study synthesises evidence from a systematic review of studies published between January 2015 and December 2025. Relevant articles were identified through searches of PubMed, Scopus, Web of Science, and Embase using targeted keywords, including “functional food”, “epigenetics”, “adolescence”, “metabolic health”, “nutrigenomics”, “obesity prevention”, and “DNA methylation”. Additional records were retrieved through manual screening of the reference lists of eligible articles to ensure comprehensive coverage of the literature. Only peer-reviewed studies published in English and involving adolescents aged 10–19 years were considered eligible. The included interventions encompassed a broad range of dietary exposures, including the consumption of fruits, vegetables, fish, meat, dairy products, cereals, plant fibre, carbohydrates, fatty acids, and sugars; comparisons of high versus low nutrient intake; and analyses of healthy versus unhealthy dietary patterns characteristic of this age group. Eligible outcomes comprised epigenetic mechanisms involved in gene regulation, including DNA methylation, histone modifications (acetylation, methylation, and phosphorylation), and non-coding RNAs such as microRNAs. Studies that did not evaluate the relationship between nutrition, epigenetic modifications, and metabolic health in adolescents were excluded.

This review is novel in its explicit integration of three domains that are rarely examined collectively: (1) functional foods and their bioactive constituents, (2) epigenetic regulatory mechanisms, and (3) metabolic health outcomes, particularly in adolescents. While previous reviews have explored diet and epigenome interactions in adults or during early developmental stages, the adolescent period—characterised by heightened epigenetic sensitivity and rapid metabolic remodelling—has received comparatively limited attention. By systematically mapping the available evidence, this review provides a critical, developmentally informed perspective on how functional foods may influence obesity risk, insulin sensitivity, lipid metabolism, and inflammatory processes through the modulation of epigenetic pathways during adolescence. This integrative approach is particularly timely given the rising prevalence of metabolic disorders among young people and the growing recognition that nutritional exposures in early life can shape long-term disease susceptibility [15,16].

The aim of this narrative review is to critically evaluate and synthesise current evidence on the epigenetic mechanisms through which functional foods and their bioactive components influence metabolic health in adolescents aged 10–19 years. Specifically, this review seeks to identify dietary exposures capable of modulating DNA methylation, histone modifications, and non-coding RNA expression in this population, and to assess the extent to which such epigenetic alterations are associated with key metabolic outcomes, including obesity, insulin resistance, dyslipidaemia, and low-grade inflammation. In addition, the methodological strengths and limitations of existing studies are examined, with particular attention to heterogeneity in dietary assessment, epigenetic measurement techniques, and study design. Finally, major gaps in the literature are highlighted, and concrete directions for future mechanistic and interventional research are proposed to clarify how functional foods may shape metabolic trajectories during this critical developmental period.

## 2. Metabolic Diseases in Adolescents During Puberty

### 2.1. Epidemiology and Core Determinants of Metabolic Syndrome in Young People

Metabolic syndrome (MetS) is a cluster of interconnected metabolic abnormalities, including central obesity, dyslipidaemia, elevated blood pressure, and impaired glucose regulation, that collectively increase the risk of cardiovascular disease and type 2 diabetes (T2DM) [14]. Importantly, MetS is defined according to heterogeneous diagnostic criteria proposed by major organisations, including the World Health Organisation (WHO), the International Diabetes Federation (IDF), and the National Cholesterol Education Program Adult Treatment Panel III (NCEP ATP III), as well as various regional guidelines. This lack of uniformity results in substantial variability in reported prevalence rates [31]. Although the specific components differ slightly across definitions, all frameworks encompass a core set of metabolic and cardiometabolic risk factors. Global estimates suggest that MetS affects approximately 20–25% of the adult population; however, prevalence varies considerably depending on the diagnostic criteria applied [31]. For example, in China, prevalence ranges from approximately 22% to nearly 39%, depending on whether WHO, IDF, or ATP III criteria are used. Comparable heterogeneity has been reported across Europe, Africa, and South Asia [31]. This definitional variability underscores the complexity of MetS and limits the comparability of epidemiological findings across studies.

Although MetS was historically regarded as a disorder of adulthood, accumulating evidence indicates that it is not age-restricted and can affect individuals across the lifespan. Its prevalence is increasing among children and adolescents, reflecting a broader deterioration in global metabolic health [32,33]. Diagnosing MetS in young populations remains challenging due to the absence of universally accepted paediatric criteria—a limitation repeatedly highlighted in the literature [34]. Despite this diagnostic ambiguity, epidemiological data consistently demonstrate that the prevalence of MetS in younger populations is rising in parallel with the global obesity epidemic.

The magnitude of this trend is striking. In 2020, MetS affected an estimated 25.8 million children (2.8%) and 35.5 million adolescents (4.8%) worldwide. Andre Pascal Kengne Noubiap et al. [35] conducted one of the most comprehensive global assessments to date, revealing marked socioeconomic and geographic disparities. The highest prevalence among adolescents (7.0%) was observed in low-income countries, whereas the greatest burden among children was reported in upper-middle-income (3.1%) and low-income regions (3.5%). Regional variation was equally pronounced: Central Latin America showed the highest prevalence in children (8.2%), while high-income English-speaking countries reported the highest rates among adolescents (6.7%). Countries such as Mexico, Spain, and Iran consistently ranked among those with the highest estimated prevalence, underscoring the complex interplay between cultural dietary patterns, socioeconomic transitions, and metabolic risk.

A consistent finding across studies is the strong association between obesity and MetS. Nearly 90% of children and adolescents with obesity exhibit at least one component of MetS, a pattern observed across diverse populations. Al-Hamad and Raman [36] report disproportionately higher prevalence rates among Hispanic youth compared with their Caucasian and African American peers, mirroring trends documented in adults of East Asian, Indian, Native American, Japanese American, and Latino descent. These ethnic disparities suggest that genetic susceptibility interacts with environmental exposures to amplify metabolic vulnerability. Evidence further indicates that mutations in the leptin (LEP) and leptin receptor (LEPR) genes demonstrate significant ethnic clustering. This includes the high prevalence of the ΔG133 frameshift mutation in consanguineous Pakistani families, as well as additional population-specific variants identified in Turkey, Turkmenistan, Egypt, Austria, and China [37,38,39,40]. Similarly, variants in the pro-opiomelanocortin (POMC) gene, a key regulator of the melanocortin appetite-suppression pathway, differ substantially across European, African, and Asian populations and exhibit ethnicity-dependent penetrance with distinct phenotypic manifestations [41,42,43,44]. These findings align with broader evidence indicating that genetic predisposition to obesity varies considerably across ethnic groups due to differences in allele frequencies, effect sizes, and gene–environment interactions [45]. This topic will be examined in greater detail in a dedicated subsection below.

Longitudinal data reinforce the clinical significance of early-life MetS. Children diagnosed with MetS are substantially more likely to develop the syndrome in adulthood and face elevated lifetime risks of T2DM and cardiovascular disease (CVD) [36]. Current estimates indicate that MetS affects approximately 10% of adolescents, with abdominal obesity being its most prevalent component. Importantly, Summer et al. [46] demonstrate that healthy dietary patterns and regular physical activity are inversely associated with central adiposity, consistent with broader evidence linking lifestyle behaviours to cardiometabolic risk.

Epidemiological studies further show that chronic diseases, including cardiovascular, metabolic, and degenerative conditions, share multiple lifestyle-related risk factors, such as smoking, unhealthy diet, obesity, and physical inactivity [47]. Notably, these factors often act synergistically. Lee et al. [48] and Beltran et al. [49] independently report that the combined presence of multiple unhealthy behaviours exerts a greater effect on disease risk than the sum of individual factors. Conversely, regular physical activity provides broad protective effects, reducing not only the incidence of MetS and coronary heart disease (CHD) but also neurological and oncological disorders [50,51].

When examining body composition, Jukarainen et al. [52] highlight that fat mass index (FMI) is more strongly associated with impaired metabolic health than either cardiorespiratory fitness (CRF) or fat-free mass index (FFMI). This finding suggests that, despite the well-documented benefits of physical fitness, adiposity itself remains a primary driver of metabolic dysfunction in young people, even when genetic and environmental influences are considered.

The pathogenesis of MetS is mechanistically complex, reflecting the interplay of genetic predisposition, insulin resistance, and environmental factors such as high-calorie diets and sedentary behaviour [34]. Pereira and Oliveira [53] further emphasise that dietary patterns established in childhood often persist into adulthood, thereby reinforcing long-term metabolic trajectories. This continuity underscores the importance of early dietary and lifestyle interventions, especially during adolescence, a period characterised by heightened biological plasticity and increased vulnerability to environmental influences. Table 1 summarises the prevalence, demographic characteristics, and key clinical components of major metabolic disorders reported in pediatric and young adult populations across various countries.

Thus, the evidence paints a clear and concerning picture: adolescence is a critical period during which metabolic risk accumulates rapidly, shaped by a combination of biological, behavioural, and environmental factors. The rising global burden of MetS in young people, together with its strong associations with obesity, lifestyle behaviours, and long-term cardiometabolic outcomes, underscores the urgent need to elucidate both the clinical manifestations and the underlying molecular mechanisms.

This context sets the stage for the following section, which moves beyond epidemiology to examine how global dietary shifts interact with the adolescent epigenome, creating a landscape of heightened vulnerability and opportunity for metabolic programming.

### 2.2. Obesity, Epigenetic Programming, and Metabolic Comorbidities

Obesity is now recognised as a chronic metabolic disease characterised by the excessive accumulation of adipose tissue. It represents one of the leading global causes of disability and premature mortality, affecting both adults and young people [60]. During adolescence, a period of rapid hormonal, neurological, and behavioural changes, obesity emerges as a potent driver of metabolic disturbances [61]. Excess adiposity promotes insulin resistance (IR), a key mechanism underlying various metabolic disorders, including MetS, T2DM, non-alcoholic fatty liver disease (NAFLD), and dyslipidaemia [62].

Modern lifestyle patterns have accelerated this trend. Increased screen time, reduced physical activity, and widespread consumption of ultra-processed foods have all contributed significantly to the rise in childhood obesity [63]. However, lifestyle factors alone cannot fully explain the rapid escalation of obesity among young people. A growing body of evidence highlights the importance of epigenetic programming, particularly during sensitive developmental periods. Early life, especially the prenatal and infancy stages, represents a critical window during which environmental exposures shape epigenetic mechanisms that influence lifelong metabolic susceptibility. This concept forms the basis of the Developmental Origins of Health and Disease (DOHaD) framework, which posits that prenatal and early postnatal environments program chronic disease risk through persistent epigenetic modifications [64].

Beyond these specific genes, numerous loci implicated in metabolic regulation exhibit obesity-related epigenetic alterations. These include tumour necrosis factor (TNF), hypoxia-inducible factor 3A (HIF3A), neuropeptide Y (NPY), insulin receptor substrate 1 (IRS1), mitochondrial transcription factor A (TFAM), interleukin-6 (IL6), lymphocyte antigen 86 (LY86), and glucose transporter type 4 (GLUT4) [65,66]. Many of these genes play key roles in inflammatory signalling, energy homeostasis, and insulin sensitivity, underscoring the molecular interconnectedness of obesity and metabolic dysfunction.

Table 2 provides an overview of the prevalence, nutritional and environmental determinants, epigenetic mechanisms, and clinical consequences of major metabolic and cardiometabolic disorders in adolescents.

Epigenetic regulation in obesity extends beyond DNA methylation. Another major layer of control involves histone modifications, such as acetylation and methylation, which act as molecular switches determining whether key adipogenic genes are activated or repressed. These dynamic histone marks orchestrate the transcription of central regulators of adipocyte differentiation, including C/EBPβ, Pref-1, aP2, peroxisome proliferator-activated receptor gamma (PPARγ), and C/EBPα—genes that collectively govern the trajectory from preadipocyte to mature adipocyte. The enzymes responsible for writing and erasing these marks, such as histone acetyltransferases (HATs), histone deacetylases (HDACs), and histone methyltransferases, serve as biochemical interpreters of the environment, translating external cues such as dietary composition, physical activity, psychosocial stress, and circadian disruption into gene expression programmes that regulate adipose tissue expansion and metabolic function [72].

This mechanistic insight highlights a central principle in obesity research: environmental exposures do not merely influence behaviour or energy balance—they directly reshape the epigenetic landscape of adipose tissue. Given that adolescence is a period of heightened hormonal flux and epigenetic plasticity, the effects of these environmentally driven histone modifications may be particularly pronounced and long-lasting, predisposing young people to accelerated adipogenesis and persistent metabolic vulnerability.

The gut microbiota adds yet another layer of complexity. Microbial metabolites, including short-chain fatty acids, can induce epigenetic modifications and modulate pathways governing appetite, lipogenesis, gluconeogenesis, inflammation, and overall metabolic regulation [73]. This suggests that obesity is not solely a host-driven process, but rather emerges from a dynamic interplay between host genetics, epigenetics, and microbial ecology.

Obesity is also characterised by chronic low-grade inflammation, driven by increased secretion of pro-inflammatory cytokines such as interleukin-6 (IL-6), tumour necrosis factor-alpha (TNF-α), and C-reactive protein (CRP). Elevated CRP levels strongly correlate with insulin resistance in overweight and obese youth [36]. This inflammatory state contributes to the development of dyslipidaemia, hypertension, and endothelial dysfunction—conditions that often emerge during adolescence and can persist into adulthood.

Dyslipidaemia, defined by quantitative abnormalities in cholesterol, lipoproteins, or triglycerides, is increasingly prevalent in children and adolescents, largely driven by obesity and associated cardiometabolic risk factors [74]. Mixed dyslipidaemia, characterised by elevated triglycerides and reduced high-density lipoprotein (HDL) cholesterol, affects 30–50% of obese adolescents [75]. Early-life epigenetic modifications may contribute to dyslipidaemia and long-term metabolic programming [76], supporting the notion that metabolic risk is established well before clinical symptoms appear.

Obesity also intersects with other metabolic comorbidities. Type 1 diabetes (T1D), a chronic autoimmune disease involving the destruction of pancreatic β-cells, is influenced by genetic, epigenetic, and environmental factors [77]. Overweight and obesity are becoming increasingly common among young people with T1D, driven by factors such as exogenous insulin therapy, reduced physical activity, fear of hypoglycaemia, and emotional eating [78]. Prenatal exposure to maternal obesity or diabetes alters foetal metabolic programming, increasing the risk of obesity, MetS, and T2DM in offspring [78,79]. Similar associations have been reported for paternal diabetes, with children of fathers with T1D showing a higher risk of developing the disease due to distinct epigenetic inheritance patterns [80,81].

T2DM is also associated with specific epigenetic alterations, including differential methylation of genes regulating insulin secretion, energy metabolism, and adipocyte function [82,83]. Collectively, these findings emphasise the pivotal role of epigenetic dysregulation in the pathogenesis of both autoimmune and metabolic forms of diabetes.

NAFLD affects up to 40% of obese adolescents and approximately 10% of children and adolescents in the general population [84]. Its pathogenesis is multifactorial, with central obesity playing a dominant role [85]. Epigenetic signatures in genes such as ankyrin-1 (*ANK1*) and microRNA-10a (*MIR10A*) have been identified in adolescents with NAFLD, irrespective of obesity status [21]. Dietary factors, including high fructose and saturated fat intake, low fibre consumption, oxidative stress, and alterations in the gut microbiome, contribute to disease progression [86,87,88].

Polycystic ovary syndrome (PCOS), which affects 5–10% of women of reproductive age and is increasingly diagnosed in adolescents, is closely associated with insulin resistance, dyslipidaemia, obesity, and elevated cardiometabolic risk [36,89]. Similarly, secondary osteoporosis is becoming more prevalent in young people, driven by nutritional deficiencies, physical inactivity, and epigenetic alterations affecting bone metabolism [90,91].

### 2.3. Epigenetic Transmission of Obesity Risk Across Generations

A substantial body of research demonstrates that parental metabolic status exerts a profound epigenetic influence on their offspring. Maternal obesity and metabolic dysfunction, for example, can alter DNA methylation of key adipokine genes, such as leptin (*LEP*) and adiponectin (*ADIPOQ*), thereby affecting adipose tissue development and metabolic programming [92]. Paternal obesity also contributes to epigenetic inheritance; reduced methylation of insulin-like growth factor 2 (IGF2) regulatory regions has been observed in the offspring of obese fathers, indicating that paternal metabolic health can shape cell growth and metabolic trajectories [72]. These findings challenge the prior assumption that epigenetic inheritance is predominantly maternal and highlight the bidirectional nature of parental contributions.

**Figure 1:**
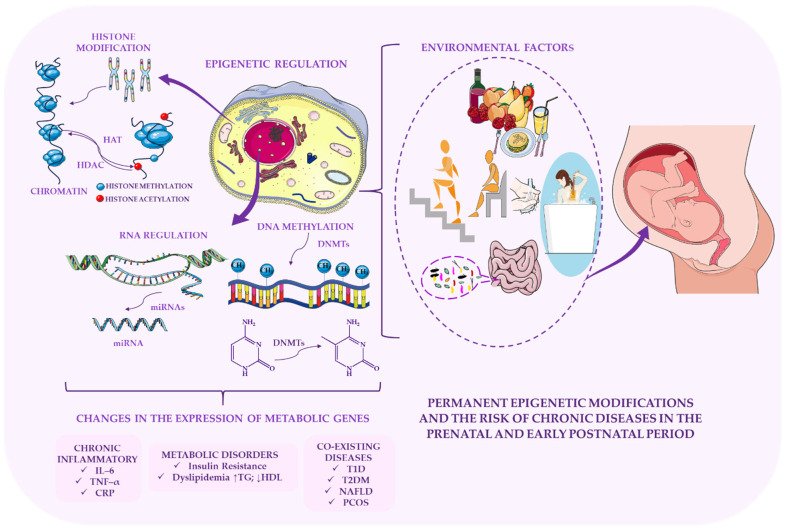
Risk of chronic diseases through prenatal epigenetic metabolic programming. Epigenetic regulation in obesity encompasses DNA methylation, histone modifications (methylation and acetylation), and microRNA-dependent mechanisms, which integrate environmental cues such as diet, physical activity, and metabolic status with gene expression programmes. These epigenetic alterations modulate the expression of key adipogenic and metabolic genes, promoting chronic low-grade inflammation, insulin resistance, and dyslipidaemia, thereby increasing the risk of metabolic syndrome (MetS), type 2 diabetes (T2DM), type 1 diabetes (T1D), non-alcoholic fatty liver disease (NAFLD), and polycystic ovary syndrome (PCOS). Image provided by Servier Medical Art (https://smart.servier.com/) (accessed on 5 January 2026), licensed under CC BY 4.0 (https://creativecommons.org/licenses/by/4.0/, accessed on 5 January 2026). Abbreviations: CRP—C-reactive protein; DNA—deoxyribonucleic acid; DNMTs—DNA methyltransferases; HAT—histone acetyltransferase; HDAC—histone deacetylase; HDL—high-density lipoprotein; IL-6—interleukin-6; miRNA—microRNA; NAFLD—non-alcoholic fatty liver disease; PCOS—polycystic ovary syndrome; RNA—ribonucleic acid; T1D—type 1 diabetes; T2DM—type 2 diabetes mellitus; TG—triglycerides; TNF-α—tumor necrosis factor alpha.

Poor maternal nutrition during the prenatal and early postnatal periods can induce long-lasting epigenetic changes that increase the risk of obesity, metabolic disorders, and cardiovascular disease (CVD) in subsequent generations [93,94,95]. Figure 1 illustrates how chronic diseases may originate from epigenetically mediated metabolic programming during the prenatal period, emphasising the enduring impact of early-life molecular imprinting on later health. Adverse childhood experiences (ACEs), including trauma, neglect, and chronic stress, have also been linked to epigenetic alterations and unhealthy eating behaviours [59,96,97]. These psychosocial factors interact with diet and physical activity to further modulate epigenetic patterns associated with metabolic dysfunction [93].

### 2.4. Ethnic Variation in LEP, LEPR and POMC Regulation and Its Relevance for Adolescents

Leptin (LEP), the leptin receptor (LEPR), and pro-opiomelanocortin (POMC) are central components of the hypothalamic appetite regulation system. Disruptions in these pathways can significantly affect body weight and metabolic risk during adolescence [37]. LEP, a hormone produced by adipose tissue, signals satiety to the brain, whereas LEPR mediates this signal in hypothalamic neurons [38]. POMC, expressed in the pituitary and hypothalamus, is a precursor protein whose cleavage products, including α-melanocyte-stimulating hormone (α-MSH), activate melanocortin receptors to suppress appetite [42]. During adolescence, a period of rapid hormonal, metabolic, and epigenetic remodelling, genetic or epigenetic alterations in these genes can have long-lasting effects on energy balance and obesity risk [39]. Ethnic disparities in obesity prevalence underscore the importance of these pathways. Substantial differences have been documented across populations living in similar environments [98], reflecting socioeconomic, lifestyle, and ancestry-specific biological factors, including variation in appetite- and adiposity-regulating genes [99].

Mutations in LEP and LEPR exhibit notable ethnic clustering. The ΔG133 frameshift mutation in LEP is prevalent among consanguineous Pakistani families [37,38,39], while other LEP variants have been reported in Turkey, Turkmenistan, Egypt, Austria, and China [40]. LEPR deficiency shows population-specific founder effects, with mutations identified in cohorts from Algeria, Bangladesh, Turkey, Iran, Southern Europe, Turkmenistan, Egypt, and Réunion Island [100,101,102,103]. Similarly, POMC mutations, including the R236G variant, vary across Danish, British, French, Italian, German, Egyptian, Indian, Turkish, and North African populations. Some groups exhibit atypical phenotypes, such as preserved pigmentation despite POMC deficiency, suggesting that penetrance and epistatic interactions are ethnicity-dependent [41,42,43,44].

These patterns align with epidemiological evidence indicating significant ethnic disparities in obesity prevalence in the United States and globally [98,99,104]. Moreover, genome-wide association studies of adiposity have been heavily biased towards European populations [105,106], with few studies conducted in African-derived cohorts [107]. Notably, variants in appetite-regulating genes have been associated with body mass index (BMI) in Black South African adolescents, highlighting the importance of ancestry-specific biology during adolescence [108]. Overall, these findings support the broader view that genetic predisposition to obesity varies substantially across ethnic groups, shaped by differences in allele frequencies, effect sizes, and gene–environment interactions [45].

### 2.5. Diet, Inflammation and Long-Term Cardiometabolic Risk

A growing body of evidence demonstrates that cardiometabolic disease begins much earlier than previously thought. Atherosclerotic changes, once considered exclusive to adulthood, can now be detected in adolescents, suggesting that vascular injury accumulates silently from a young age. Adipose tissue dysfunction related to obesity plays a central role in this early pathogenesis by promoting the secretion of pro-inflammatory, diabetogenic, and atherogenic mediators [109]. These factors, including interleukins, TNF-α, and adipokines, create a chronic inflammatory environment that accelerates endothelial damage and metabolic deterioration.

Dietary patterns can significantly influence this inflammatory state. High-fat diets (HFDs), for example, have been shown to induce epigenetic dysregulation, affecting gene transcription, energy homeostasis, hormonal signalling, and inflammatory pathways [110,111]. These effects are not limited to macronutrient composition; micronutrient availability also shapes the epigenome. Fluctuations in methyl donors, particularly the S-adenosylmethionine/S-adenosylhomocysteine (SAM/SAH) ratio, as well as B vitamins, choline, and fatty acids, influence DNA methylation and histone modifications, thereby altering the expression of metabolic genes [112]. Together, these findings emphasise diet as a molecular signal capable of reprogramming metabolic pathways.

Not all dietary components exert uniformly detrimental effects. Akbary Sedigh et al. [113] reported that calcium and dairy intake during adolescence do not increase carotid intima-media thickness (cIMT), a marker of subclinical atherosclerosis, nor do they elevate the risk of MetS in early adulthood. However, the same study found that long-term high intake of total calcium or low-fat dairy may increase triglyceride levels and diastolic blood pressure, suggesting that the cardiometabolic impact of dairy depends on dose, fat content, and dietary context. This contrasts with the more uniformly adverse effects of HFDs, highlighting the importance of distinguishing between dietary patterns rather than broadly categorising foods as “good” or “bad”.

Obesity remains one of the strongest drivers of early cardiometabolic dysfunction. Paediatric hypertension, affecting 3–5% of children and adolescents, is closely linked to excess adiposity [114]. Diagnostic criteria rely on age-specific percentiles up to age 16, after which adult thresholds are applied [115]. Poor diet quality exacerbates this risk; up to 41% of adolescents consume low-quality diets characterised by high sugar intake, low fibre, and insufficient micronutrients [116]. These dietary patterns promote weight gain, intensify inflammation and oxidative stress, and create a self-reinforcing cycle of metabolic impairment.

Rai (2024) [112] demonstrated that exposure to palmitate, a key component of high-fat diets, and related metabolites such as acetyl-CoA, succinate, and α-ketoglutarate can induce developmental programming of atherosclerosis. These metabolic signals were shown to alter epigenetic regulation, including DNA methylation of genes such as *SETD2*, *IRS2*, and *MAP2K4*, as well as histone acetylation and succinylation, alongside dysregulation of non-coding RNAs. Such epigenetic reprogramming was associated with increased susceptibility to obesity, insulin resistance, glucose intolerance, T2DM, NAFLD, and cardiomyopathy in the offspring [112].

The anti-inflammatory potential of certain dietary components offers a promising counterbalance. Polyphenols, bioactive compounds found in fruits, vegetables, tea, and cocoa, exert potent anti-inflammatory and antioxidant effects that may mitigate MetS-related inflammation [117]. These mechanisms include inhibition of NF-κB signalling, reduction in oxidative stress, and modulation of adipokine secretion. Moreover, diet influences epigenetic markers, including DNA methylation, histone remodelling, and non-coding RNAs, collectively regulating the expression of inflammatory genes [118]. These epigenetic effects highlight the potential for dietary interventions to reverse or attenuate pathological processes, particularly during adolescence, a period of heightened epigenetic plasticity.

Thus, poor dietary patterns during adolescence are not merely a short-term health concern but constitute a significant risk factor for metabolic disease later in life. The convergence of inflammation, epigenetic dysregulation, and unhealthy dietary exposures during this critical developmental period underscores the importance of early nutritional and educational interventions. By shaping the epigenome and inflammatory pathways, adolescent diet exerts a profound influence on long-term cardiometabolic health.

## 3. Global Dietary Shifts and Epigenetic Vulnerability in Youth

An increasing body of evidence suggests that rapid global changes in diet and lifestyle are reshaping the metabolic and epigenetic landscape of young people, with early-life nutritional exposures exerting long-lasting biological effects [61,119,120]. As Barbalho et al. [61] note, global economic growth has profoundly influenced dietary habits and physical activity, contributing to a marked rise in overweight, obesity, and cardiovascular disease. This phenomenon represents not only a public health concern but also a broader societal challenge, disproportionately affecting children, adolescents, and low-income families over the past decade.

**Figure 2:**
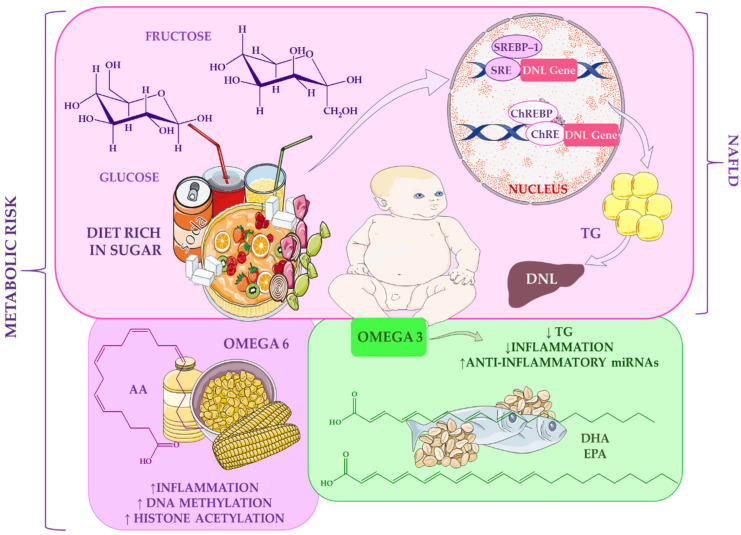
Dietary sugars and fatty acids in the regulation of metabolic risk. Fructose and glucose both activate carbohydrate-responsive element-binding protein (ChREBP); however, fructose additionally stimulates sterol regulatory element-binding protein 1 (SREBP-1), leading to a stronger induction of lipogenic gene expression and reduced fatty acid oxidation. As a result, diets high in these sugars promote hepatic lipid accumulation and increase the risk of non-alcoholic fatty liver disease (NAFLD). In contrast, omega-3 fatty acids, including docosahexaenoic acid (DHA) and eicosapentaenoic acid (EPA), exert protective metabolic effects by reducing inflammation, dyslipidaemia, and insulin resistance. Conversely, omega-6 fatty acids, particularly arachidonic acid, are associated with increased metabolic risk, in part through epigenetic regulation involving DNA methylation and histone modifications. Image provided by Servier Medical Art (https://smart.servier.com/) (accessed on 5 January 2026), licensed under CC BY 4.0 (https://creativecommons.org/licenses/by/4.0/, accessed on 5 January 2026). Abbreviations: ChRE—carbohydrate response element; ChREBP—carbohydrate-responsive element-binding protein; DHA—docosahexaenoic acid; DNA—deoxyribonucleic acid; DNL—*de novo* lipogenesis; EPA—eicosapentaenoic acid; SRE—sterol regulatory element; SREBP-1—sterol regulatory element-binding protein 1; TG—triglycerides. ↑—increase in activity/level/expression; ↓—decrease in activity/level/expression.

The significance of these findings lies in the demonstration that economic transitions and dietary shifts intersect to create an environment conducive to early-onset metabolic disease. Importantly, such environmental pressures interact with biological systems during critical developmental periods, amplifying long-term metabolic risk. Figure 2 illustrates how dietary sugars and fatty acids influence metabolic risk, highlighting their interconnected roles in shaping energy homeostasis, insulin sensitivity, and susceptibility to metabolic disorders over the lifespan.

Rubinstein and Low [119] further emphasise that modern lifestyle changes affect individuals differently, with some being more susceptible to obesity, suggesting underlying biological and epigenetic differences in metabolic regulation. This observation is important because it indicates that environmental exposure alone cannot fully explain the obesity epidemic; gene–environment interactions, including diet-induced epigenetic modifications, also play a decisive role. At the molecular level, dietary excess and sedentary behaviour can alter pathways such as insulin signalling, adipokine secretion, and hypothalamic appetite regulation. All of these pathways are sensitive to epigenetic modulation via DNA methylation, histone modifications, and microRNA expression. Rubinstein and Low’s work, therefore, highlights the need to consider both environmental and molecular determinants of metabolic vulnerability.

The consumption of ultra-processed foods (UPFs) is increasing rapidly, as noted by Elizabeth et al. [120]. These industrial formulations are predominantly composed of refined sugars, oils, starches, and isolated proteins, with minimal whole-food content, and often contain additives to enhance palatability and shelf life. Their study demonstrates that UPFs are a major contributor to declining diet quality among children and adolescents. Crucially, these products represent an environmental factor capable of triggering adverse epigenetic changes. Diets high in refined sugars and additives can stimulate hepatic *de novo* lipogenesis, disrupt gut microbiota composition, and activate inflammatory pathways such as NF-κB and JNK. These processes can influence chromatin structure and gene expression. By linking dietary patterns to molecular alterations, Elizabeth et al. [120] provide compelling evidence that poor-quality diets during youth may establish long-lasting epigenetic signatures associated with metabolic disease.

Nutrients play a far broader role than previously recognised. Beyond their classical function as energy sources and structural components, they act as biochemical signals and potent epigenetic modulators, orchestrating DNA methylation, histone dynamics, and microRNA expression. In doing so, they shape gene activity and direct metabolic and developmental trajectories throughout life. This is particularly relevant in the context of shifting dietary patterns, as many nutrients are known to protect against chronic disease, partly through epigenetic mechanisms [121].

A variety of epigenetic modifications can be induced both prenatally and during adulthood via supplementation with nutrients such as folic acid and methionine, largely due to the influence of methyl-donating compounds on DNA methylation [122]. Key nutrients, including folate, vitamins B_12_, B_6_, and B_2_, as well as choline and betaine, serve as methyl donors or cofactors in one-carbon metabolism, a biochemical network that governs the transfer of methyl groups required for DNA and histone methylation [123].

Several nutrients and bioactive dietary compounds directly modulate the activity of enzymes responsible for epigenetic regulation, including DNA methyltransferases (DNMTs), histone acetyltransferases (HATs), and histone deacetylases (HDACs) [123]. DNA methylation, the addition of a methyl group to cytosine residues, serves as a central mechanism for regulating gene expression [122]. While traditionally associated with CpG dinucleotides, emerging evidence indicates that methylation can also occur in non-CpG contexts, underscoring its complexity and regulatory importance. The primary methyl donor in this process is S-adenosylmethionine (SAM), synthesised from methionine and utilised by DNMTs to generate 5-methylcytosine (5mC). After methyl donation, SAM is converted to S-adenosylhomocysteine (SAH), which must be remethylated to regenerate methionine via folate-dependent or choline/betaine-dependent pathways [123].

Numerous studies have demonstrated that dietary methyl donors, including folate, vitamins B_12_ and B_2_, choline, and betaine, play a central role in one-carbon metabolism. This pathway generates S-adenosylmethionine (SAM), the universal methyl donor required for DNA methylation. A key enzyme in this pathway is methylenetetrahydrofolate reductase (MTHFR), located on chromosome 1p36.6. The common C677T polymorphism reduces MTHFR activity and increases susceptibility to elevated homocysteine levels, particularly in individuals with the TT genotype [124]. Population studies consistently show that the metabolic consequences of the C677T variant are strongly influenced by dietary folate and B-vitamin status. For instance, in a study of Korean adults, individuals with the TT genotype required substantially higher folate intake to maintain normal homocysteine levels, demonstrating a clear gene–nutrient interaction [125]. Similar associations have been reported in pregnant women, where the TT genotype correlates with higher homocysteine concentrations, especially under low serum folate, vitamin B_2_, or vitamin B_12_ conditions [126]. These interactions are not restricted to East Asian populations. Among Mexican women of reproductive age, both dietary folate intake and MTHFR genotype independently influenced homocysteine status [127]. In Brazilian pregnant women, combined polymorphisms in MTHFR, MTR, and MTRR were associated with reduced B-vitamin levels and elevated homocysteine [128]. Further evidence from Chinese adults with hypertension indicates that folate, homocysteine, and polymorphisms in one-carbon metabolism genes collectively affect metabolic outcomes, including dyslipidaemia [129]. These studies underscore that nutritional epigenetic responses are strongly genotype-dependent, and populations with a higher prevalence of the MTHFR C677T variant may be more sensitive to dietary methyl donor availability.

The efficiency of one-carbon metabolism and DNA methylation thus depends heavily on the dietary availability of essential micronutrients, particularly folate, choline, betaine, and vitamins B_12_ and B_6_ [130,131]. Deficiencies in these nutrients, as well as in methionine, can significantly alter the SAM:SAH ratio, thereby affecting global DNA methylation patterns [123]. Vitamin B_12_ is especially critical because it is predominantly found in animal-derived foods; consequently, vegetarians and vegans frequently exhibit reduced serum B_12_ levels [132]. B_12_ deficiency impairs methionine synthase (MS) activity, leading to hyperhomocysteinemia and increased production of reactive oxygen species (ROS), both of which elevate cardiovascular and neurological risk [132]. Reduced MS activity also limits SAM synthesis, while concurrent accumulation of methylmalonic acid may inhibit mitochondrial electron transport. Experimental studies further demonstrate that B_12_ deficiency decreases mitochondrial gene expression and carnitine transport in intestinal cells [133], reduces microbiota-derived fatty acid production, and impairs peroxisome proliferator-activated receptor (PPAR) signalling and β-oxidation. These effects decrease oxygen consumption and promote the expansion of *Salmonella enterica*, altering its virulence gene expression [132].

Evidence from both animal and human studies indicates that high sugar consumption, particularly fructose, is a major risk factor for obesity and NAFLD in children, with adverse effects potentially beginning as early as the foetal stage [84,134,135]. Fructose potently stimulates hepatic *de novo* lipogenesis (DNL), a key driver of steatosis [84,136,137]. In mice chronically fed a high-fat diet, the addition of sugars further increased triglyceride production [138,139]. While both fructose and glucose activate the transcription factor ChREBP, fructose uniquely stimulates SREBP-1, amplifying the expression of lipogenic genes [138]. As a result, diets high in fructose and glucose suppress fatty acid oxidation and promote lipid synthesis more strongly than glucose-rich diets alone, directly increasing the risk of hepatic steatosis [139].

Nutritional epigenetics research is increasingly focusing on how dietary exposures shape epigenetic marks and cellular phenotypes, providing a foundation for precision nutrition. Systematic reviews highlight significant interactions between dietary fatty acids and epigenetic regulation [140]. Omega-3 fatty acids, including DHA and EPA, have been associated with a lower risk of metabolic disturbances, such as dyslipidaemia, inflammation, and insulin resistance. In contrast, omega-6 fatty acids, particularly arachidonic acid, have been linked to increased metabolic risk through their effects on DNA methylation, histone acetylation, and microRNA expression [140].

These findings indicate that modern dietary patterns, characterised by high intake of ultra-processed foods, sugars, and nutrient-poor diets, interact with epigenetic mechanisms that regulate metabolic health. Adequate consumption of methyl-donor nutrients and bioactive compounds is essential for maintaining proper one-carbon metabolism, DNA methylation, and mitochondrial function. As adolescence represents a period of heightened metabolic and epigenetic plasticity, tailored nutritional strategies may provide an effective means to prevent obesity, NAFLD, and cardiovascular disease throughout life.

Given the rising prevalence of obesity, insulin resistance, and NAFLD among adolescents, understanding the molecular mechanisms through which diet affects metabolic health is crucial. Epidemiological evidence suggests that environmental and nutritional exposures during this developmental window have long-lasting effects, largely mediated by epigenetic regulation. Accordingly, the following section focuses on DNA methylation, histone modifications, and microRNA dynamics as mechanistic pathways linking dietary patterns to metabolic outcomes. By integrating population-level observations with molecular evidence, we highlight how the unique epigenetic plasticity of adolescence creates a critical period during which dietary exposures can shape long-term metabolic trajectories. This framework provides a conceptual bridge between epidemiological trends and the mechanistic insights discussed in the subsequent section.

## 4. Epigenetic Plasticity During Adolescence: A Critical Window for Nutritional Programming

Several authors emphasise that adolescence represents a unique “window of epigenetic plasticity”, during which dietary exposures can permanently alter gene expression patterns and influence the long-term risk of metabolic, neurocognitive, and psychological disorders. Nicoletti et al. [123] argue that this developmental period is characterised by heightened sensitivity of the epigenome to environmental inputs, particularly nutrients involved in one-carbon metabolism and chromatin remodelling. These observations align with earlier findings by Bianco-Miotto et al. [64], who demonstrated that dynamic epigenetic remodelling during critical growth phases can establish persistent transcriptional trajectories affecting metabolic homeostasis in adulthood. Similarly, Gkiouleka et al. [141] provide evidence that lifestyle interventions introduced during adolescence, especially dietary modifications and increased physical activity, can reverse adverse epigenetic signatures associated with insulin resistance and impaired mitochondrial function.

A consistent pattern emerges across these studies: adolescence is not merely a transitional stage of physiological maturation but a period during which the epigenome remains exceptionally malleable. This plasticity allows nutritional factors to influence DNA methylation, histone modifications, and microRNA expression, potentially mitigating or exacerbating susceptibility to obesity, type 2 diabetes, and chronic low-grade inflammation. Importantly, Moormann et al. [142] emphasise that these epigenetic effects may extend intergenerationally by influencing germline epigenetic marks.

Thus, the extant evidence underscores the profound biological significance of adolescent nutrition in shaping lifelong metabolic and cognitive health. The convergence of findings across multiple research groups supports the notion that targeted nutritional strategies during this developmental window could serve as an effective approach for preventing metabolic dysfunction and promoting long-term well-being.

### 4.1. Epigenetic Mechanisms in the Context of Adolescent Plasticity

Dincer (2016) emphasises that epigenetics encompasses heritable changes in genome function that do not arise from alterations in the DNA sequence itself but rather from chemical modifications of DNA and chromatin [143]. Ostaiza-Cárdenas et al. [144] note that these modifications can be stably transmitted through successive cell divisions and are essential for the proper regulation of gene expression. Carlberg and Molnár [145] further observe that epigenetic reprogramming determines how genetic information is interpreted, thereby influencing cellular differentiation, metabolic function, and responses to environmental stimuli.

According to Dincer [143], epigenetic modifications accumulate throughout life and are shaped by environmental factors such as diet, lifestyle, and exposure to toxins. Importantly, when these modifications occur in germ cells, they may be transmitted to offspring. Cavalli and Heard [146] report that intergenerational transmission of epigenetic marks, including DNA methylation and histone modifications, can stabilise phenotypic traits induced by parental exposure to environmental stressors. This effect is observed in the first male generation and in both the first and second female generations. Moormann et al. [142] attribute this phenomenon to the direct influence of environmental factors on oocytes present in the developing female foetus.

Essential cellular processes, including genomic imprinting, X-chromosome inactivation, DNA damage responses, cellular reprogramming, and ageing, are regulated by epigenetic mechanisms [123,143]. These processes, which include DNA methylation, histone modifications, and non-coding RNA regulation, play a pivotal role in metabolic adaptation, influencing susceptibility to obesity, insulin resistance, and type 2 diabetes. Their significance is particularly pronounced during adolescence, a developmental period characterised by heightened epigenetic sensitivity to nutritional and environmental cues.

### 4.2. Epigenetic Regulation of Metabolism During Adolescence

Gkiouleka et al. [141] demonstrate that lifestyle interventions, including dietary modifications and increased physical activity, can alter epigenetic patterns even during adolescence. Specifically, they show that hypermethylation of PGC1A and PPARGC1B is associated with insulin resistance, whereas histone acetylation promotes the expression of genes involved in mitochondrial biogenesis and glucose uptake. These findings align with those of Russo et al. [20], who emphasise that epigenetic changes can be both stable and dynamic, thereby shaping metabolic phenotypes across the lifespan. In the context of adolescence, this heightened plasticity suggests that metabolic trajectories can be redirected towards healthier outcomes through appropriately targeted nutritional strategies.

### 4.3. DNA Methylation as a Central Epigenetic Mechanism

Corbin et al. [78] describe DNA methylation as a fundamental epigenetic mechanism and one of the most prevalent chemical modifications of the genome. DNA methyltransferases (DNMTs) catalyse the transfer of methyl groups to cytosine residues within CpG dinucleotides, establishing transcriptionally repressive chromatin states that regulate gene accessibility. Grazioli et al. [47] emphasise that disruptions to these methylation patterns—whether through hypermethylation of tumour-suppressor loci or global hypomethylation—can destabilise genomic integrity and contribute to the onset of metabolic and neoplastic diseases.

**Figure 3:**
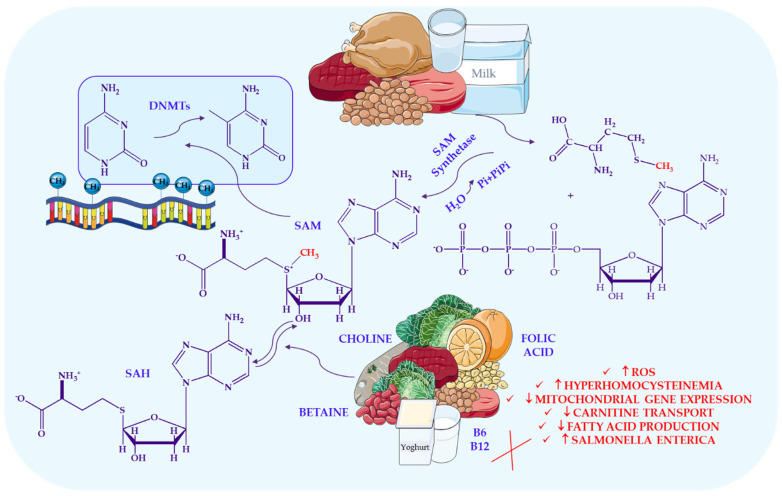
Dietary components as epigenetic modulators of DNA methylation. DNA methylation relies on the availability of S-adenosylmethionine (SAM), the universal methyl donor synthesized from methionine and utilized by DNA methyltransferases (DNMTs). After donating a methyl group, SAM is converted to S-adenosylhomocysteine (SAH), which must be remethylated to regenerate methionine via folate- or choline/betaine-dependent pathways that require vitamins B_6_ and B_12_. Deficiencies in these nutrients disrupt the SAM/SAH ratio, leading to altered global DNA methylation patterns. In particular, vitamin B_12_ deficiency impairs methionine synthase activity, promotes homocysteine accumulation, increases oxidative stress, induces mitochondrial dysfunction, and perturbs lipid metabolism and gut–host metabolic signaling. Image provided by Servier Medical Art (https://smart.servier.com/) (accessed on 5 January 2026), licensed under CC BY 4.0 (https://creativecommons.org/licenses/by/4.0/, accessed on 5 January 2026). Abbreviations: B_6_—vitamin B_6_ (pyridoxine); B_12_—vitamin B_12_ (cobalamin); DNMTs—DNA methyltransferases; P_i_—inorganic phosphate; PiPi—pyrophosphate (inorganic pyrophosphate, PPi); ROS—reactive oxygen species; SAH—S-adenosylhomocysteine. ↑—increase in activity/level/expression; ↓—decrease in activity/level/expression.

Li et al. [123,147] highlight that the fidelity of DNA methylation is closely linked to nutritional status, particularly the availability of methyl-donor nutrients such as folate, vitamins B_12_, B_6_, and B_2_, as well as choline and betaine. These compounds support one-carbon metabolism, shaping the intracellular pool of S-adenosylmethionine (SAM), the universal methyl donor required for both DNA and histone methylation. Consistent with this biochemical framework, Amenyah et al. [148,149] demonstrate that fluctuations in dietary supply of these nutrients can recalibrate methylation landscapes by modulating substrate availability or altering DNMT activity—a finding further supported by Lees-Murdock et al. [150]. Figure 3 illustrates how specific dietary elements act as epigenetic modulators of DNA methylation, highlighting their capacity to influence gene expression patterns and metabolic pathways throughout life.

Importantly, these nutrient-dependent effects are especially pronounced during adolescence, a developmental period characterised by rapid growth, hormonal reorganization, and heightened epigenomic sensitivity. The increased metabolic demand for methyl donors during this stage suggests that dietary patterns may exert a disproportionately strong and lasting influence on DNA methylation profiles. Consequently, ensuring nutritional adequacy during adolescence may play a critical role in shaping long-term metabolic programming and modulating susceptibility to conditions such as insulin resistance, obesity, and related cardiometabolic disorders.

### 4.4. Role of MicroRNAs in Epigenetic Regulation

Lorente-Cebrián et al. [151] characterised miRNAs as remarkably potent regulators of gene expression despite their small size. These short, 18–25-nucleotide RNA molecules, as noted by Lin and Li [72], can influence the expression of nearly one-third of all human genes, highlighting their broad biological impact. Yao et al. [152] further demonstrate that miRNAs operate within intricate feedback networks that interact with core epigenetic mechanisms, and Poddar et al. [153] and Jiang et al. [154] reinforce this interplay by showing that miRNA expression is itself shaped by DNA methylation and histone modifications, creating multilayered regulatory circuits.

Grazioli et al. [47] emphasise the functional reach of miRNAs, including essential roles in development, immune regulation, and cardiac remodelling, while Ji and Guo [155] and Huang et al. [156] link dysregulated miRNA profiles to obesity and diabetes. Lin and Li [72] further highlight that miRNAs orchestrate adipocyte differentiation and contribute to the chronic low-grade inflammation characteristic of obesity.

What makes miRNAs particularly interesting in adolescence is their exceptional sensitivity to dietary cues. Lorente-Cebrián et al. [151] demonstrate that polyphenols can modulate miRNAs regulating brown adipose tissue activity. Ribeiro et al. [157] show that pistachio intake alters PI3K-AKT-related miRNAs, and Assmann et al. [158] report that low-fat diets can modify the expression of obesity-associated miRNAs, such as miR-142-5p and miR-221-3p. Marques-Rocha et al. [159] additionally reveal that oleic acid can downregulate pro-inflammatory miRNAs, including let-7b.

Together, these findings underscore the capacity of adolescent dietary patterns to reprogram metabolic pathways through miRNA-mediated epigenetic mechanisms, potentially exerting long-lasting effects on metabolic health and disease susceptibility.

### 4.5. Histone Modifications and Chromatin Remodelling

The relevance of histone modifications and chromatin remodelling is particularly striking during adolescence, a period characterised by rapid cellular proliferation, hormonal reorganisation, and extensive tissue remodelling. Such intense biological activity requires highly responsive chromatin-modifying and chromatin-remodelling systems. Research shows [47,160] that histone modifications such as acetylation, methylation, phosphorylation, lactylation, and ubiquitination constitute a dynamic and versatile layer of epigenetic regulation. These chemical marks act as regulatory signals that fine-tune gene accessibility, with their effects dependent on the enzymatic systems responsible for installing or removing them.

Key enzymes include histone acetyltransferases (HATs), which add acetyl groups to lysine residues and promote open chromatin, and histone deacetylases (HDACs), which remove acetyl groups to restore chromatin compaction. Histone methyltransferases (HMTs) catalyse methylation of lysine or arginine residues, while demethylases such as LSD1 and Jumonji-domain histone demethylases (JHDMs) reverse these marks via Fe^2+^- and α-ketoglutarate-dependent mechanisms. PADI4 (peptidyl arginine deiminase 4) further diversifies regulation by converting arginine residues into citrulline, altering histone charge and chromatin accessibility. Together, these enzymes form a highly responsive network capable of rapidly translating metabolic and environmental cues into transcriptional outcomes. Di Nisio et al. [161] highlight that histone methylation can either activate or repress transcription depending on the specific residue, underscoring the precision of this regulatory mechanism.

The structural context is equally important. The nucleosome, identified by Parmar and Padinhateeri [162] as the fundamental unit of chromatin, typically imposes a barrier to transcription. ATP-dependent chromatin-remodelling complexes, such as SWI/SNF, ISWI, CHD, and INO80 [163], reposition, restructure, or evict nucleosomes to modulate DNA accessibility. Comparative analyses [164,165,166,167] reveal that these complexes differ in structure and regulatory specificity, highlighting their complementary roles in maintaining transcriptional flexibility.

These studies indicate that the adolescent epigenome is especially susceptible to environmental and nutritional influences. Histone modifications and chromatin-remodelling events established during this window can create long-lasting transcriptional programmes that shape metabolic health, neurodevelopment, and disease susceptibility well into adulthood.

## 5. Functional Foods as Epigenetic Modulators During Adolescence: Molecular Pathways, Metabolic Implications and Developmental Vulnerabilities

The term “functional foods” remains inconsistently defined across scientific and regulatory frameworks. Common descriptions, such as that of the Food and Agriculture Organization (FAO), which defines them as foods containing components beneficial to health beyond their nutrient content, and that of the Mayo Clinic, which describes them as foods exerting positive effects “beyond basic nutrition” [22], are so broad that they often encompass nearly all nutrient-dense foods, including fish, legumes, whole grains, and nuts [24]. As Temple [24] argues, this inclusiveness blurs the distinction between ordinary healthy foods and products intentionally engineered to deliver targeted physiological benefits. This becomes problematic when foods such as beetroot, peanuts, sweet potatoes, pomegranate juice, strawberries, or yoghurt are labelled as “functional” solely because they contain phytochemicals or probiotics, despite their health effects arising from complex interactions among nutrients, fibre, and bioactive compounds. The ambiguity surrounding what constitutes “beyond basic nutrition” further complicates classification, particularly when nutrients and non-nutritive phytochemicals have overlapping functions, as in the case of carotenoids. In contrast, intentionally enriched products, such as calcium-fortified juices, omega-3-enhanced margarines, foods containing added plant sterols or stanols, and formulations incorporating probiotics, prebiotics, or concentrated catechins and anthocyanins, represent clearer examples of foods designed to deliver specific physiological effects.

There is evidence supporting the functional potential of such added components. For example, oat β-glucans modulate cholesterol metabolism and microbiome-dependent bile acid pathways [168]; inulin-type fructans exert prebiotic effects on the colonic microbiota [169]; plant sterols and stanols reduce LDL-cholesterol, albeit with notable interindividual variability [170]; and tea catechins influence metabolic and cardiovascular pathways [171]. Similarly, anthocyanin-rich berries demonstrate benefits for metabolic syndrome, antiviral defence, digestive and immune function, and chronic disease prevention [172,173,174,175,176]. However, even calcium-fortified beverages, though widely consumed, remain the subject of debate regarding their long-term benefits and safety [177].

To address these discrepancies, Temple [24] proposes a more stringent definition of functional foods as novel formulations containing added bioactive substances or live microorganisms at concentrations that are both safe and sufficiently high to produce measurable health-enhancing or disease-preventive effects. This perspective aligns with definitions proposed by the Institute of Food Technologists [25] and the Functional Food Centre, which emphasise intentional formulation, standardised bioactive content, and demonstrable physiological benefits as defining characteristics of functional foods [27,28,29]. Recent clinical research further supports the potential of such engineered products to improve metabolic, cardiovascular, and immune outcomes [30].

Because functional foods may represent a valuable nutritional strategy during adolescence, a developmental period marked by rapid somatic growth, hormonal reorganisation, and heightened metabolic vulnerability, their role warrants careful consideration in this context. The bioactive components of functional foods, including vitamins, polyphenols, probiotics, prebiotics, and unsaturated fatty acids, can support immune maturation, stabilise the gut microbiome, and modulate key molecular pathways such as AMPK, PPARγ, NF-κB, and mTOR. Collectively, these pathways regulate energy homeostasis, inflammatory tone, and metabolic adaptation.

Table 3 provides a comprehensive overview of key epigenetic modulators present in functional foods, highlighting specific bioactive compounds, their molecular targets, and the downstream metabolic adaptations they induce. As illustrated in Table 3, numerous bioactive compounds derived from functional foods exert measurable epigenetic effects, including modulation of DNMT and HDAC activity, histone remodelling, and alterations in metabolic gene expression.

However, the incorporation of functional foods into an adolescent’s diet should be considered within a broader lifestyle framework encompassing balanced nutrition, regular physical activity, and adequate sleep hygiene. Given that adolescents often rely on marketing cues rather than nutritional value when making dietary choices, educational guidance is essential to promote informed decision-making and prevent misconceptions regarding the role of functional foods.

### 5.1. Endocrine–Neurobiological Vulnerability of Adolescence

Adolescence is a developmental period characterised by exceptional physiological plasticity and increased susceptibility to internal and external stressors that can disrupt the tightly regulated processes of growth and maturation. The rate and quality of somatic development depend on the dynamic interplay among genetic predispositions, environmental exposures, hormonal signalling, and nutritional status. Chronic activation of the stress axis, manifested as sustained hypercortisolaemia, suppresses the secretion of growth hormone (GH), insulin-like growth factor 1 (IGF-1), and thyroid and sex steroids. This may lead to a range of adverse consequences, including growth deceleration, increased visceral adiposity, reduced muscle and bone mass, and the development of insulin resistance. Furthermore, glucocorticoids antagonise the anabolic effects of GH and gonadal hormones by inhibiting lipolysis and protein synthesis. Prolonged dysregulation of the hypothalamic–pituitary–adrenal axis can result in symptoms resembling Cushing’s or pseudo-Cushing’s syndrome and contribute to osteoporosis and broader metabolic disturbances [191].

This complexity warrants emphasis, as it underscores that adolescence is not merely a transitional stage but a period during which endocrine and metabolic systems are exquisitely sensitive to nutritional modulation. It therefore represents both a uniquely opportune and a potentially precarious window for interventions aimed at shaping long-term metabolic health. Understanding this endocrine vulnerability is essential, because the hormonal milieu of adolescence does not operate in isolation; rather, it is embedded within a broader neurobiological framework that continuously integrates metabolic cues from peripheral tissues, thereby linking nutritional exposures to central regulatory mechanisms.

In vertebrates, body weight and energy balance are regulated by highly specialised neural circuits that integrate metabolic, hormonal, and environmental signals with current and anticipated energetic demands, shaping adaptive behaviours that range from foraging and food intake to satiety. Peripheral organs, including adipose tissue, the pancreas, liver, and gastrointestinal tract, play an indispensable role in this process by releasing hormones and metabolites in response to nutrient flux. Circulating information regarding the organism’s energetic state is subsequently decoded by distinct neuronal populations located primarily in the arcuate nucleus of the hypothalamus and the brainstem, which together form a central integrative hub for metabolic regulation [119].

This neuroendocrine integration is particularly relevant during adolescence, as it illustrates how dietary patterns and nutrient composition can directly influence central appetite regulation, energy expenditure, and metabolic homeostasis. It also provides a mechanistic foundation for understanding how functional foods and their bioactive components may exert epigenetic and metabolic effects during this critical developmental stage.

### 5.2. Adipose Tissue as an Endocrine and Epigenetically Responsive Organ and Implications for Adolescent Metabolic Health

Adipose tissue, as emphasised by An et al. [192], modulates systemic metabolism not only through the uptake and storage of glucose and fatty acids but also through the secretion of an exceptionally diverse repertoire of bioactive molecules, including hormones, metabolites, and extracellular genetic material. This perspective expands earlier, more reductionist views of adipocytes as mere lipid reservoirs and aligns with the broader conceptual shift recognising adipose tissue as a complex endocrine organ. Although An and colleagues highlight that skeletal muscle accounts for the majority (80–85%) of insulin-stimulated glucose uptake, they also demonstrate that adipose tissue expresses insulin-regulated GLUT4 and therefore contributes meaningfully to peripheral glucose clearance. This finding complements the work of Carson et al. [193], who show that thermogenic adipose depots, particularly brown and beige adipocytes, can act as potent glucose sinks under adrenergic stimulation. Together, these observations suggest that distinct adipose depots possess different metabolic capacities and may respond differentially to dietary or environmental modulation.

Recognising adipose tissue as a dynamic endocrine organ, as emphasised by An et al. [192], underscores that specific adipocyte-derived hormones, most notably leptin and adiponectin, serve as molecular bridges linking nutritional status, energy homeostasis, and metabolic risk. This contrasts sharply with earlier models portraying adipose tissue as a passive lipid depot and aligns with contemporary evidence positioning it as a metabolically active, signalling-rich organ. According to Rubinstein and Low [119], leptin functions as a circulating indicator of stored energy. In contrast, Ghadge and Khaire [194] emphasise its broader role in glucose and lipid metabolism, illustrating how different research groups approach the same hormone from complementary mechanistic perspectives. This plurality of perspectives is significant because it reinforces the concept that adipokines are central regulators rather than mere metabolic by-products, and that their secretion and downstream signalling are sensitive to dietary composition. This is particularly relevant in adolescence, a developmental stage characterised by heightened metabolic plasticity, when nutritional exposures may influence endocrine signalling and epigenetic pathways with long-term consequences for metabolic health.

The molecular cloning of the leptin gene, first demonstrated in foundational genetic studies and subsequently expanded upon by Mousikou et al. [191], revealed that mutations in this gene underlie an autosomal recessive form of early-onset severe obesity. This discovery accelerated the identification of hypothalamic circuits and genes directly involved in the central regulation of food intake. Mousikou et al. [191] further demonstrate that leptin stimulates GH secretion through hypothalamic mechanisms. Rubinstein and Low [119], however, emphasise its function as a continuous biochemical readout of triglyceride stores, illustrating how the same hormone may be conceptualised either as a metabolic sensor or as a neuroendocrine regulator. Tragomalou et al. [195] extend this framework by showing that both leptin and adiponectin play pivotal roles in the pathogenesis of metabolic syndrome, whereas Ghadge et al. [196] focus on their involvement in energy expenditure and inflammatory regulation, underscoring the multidimensional nature of adipocytokine function.

Yanai and Yoshida [197] describe the anti-inflammatory and anti-atherogenic properties of adiponectin, while Tragomalou et al. [195] emphasise its cardioprotective effects. These differences illustrate how individual authors prioritise distinct physiological outcomes. In contrast, Javier et al. [198] report no significant differences in adiponectin levels among children with metabolic syndrome, a finding that diverges from earlier paediatric studies and may reflect methodological variability, developmental stage, or sample heterogeneity. Francisco et al. [199] propose that leptin levels exceeding 13.4 ng/dL may indicate an increased risk of metabolic syndrome in prepubertal children. Saklayen [200] further notes that adipocytes secrete more than a dozen hormones influencing appetite and satiety, and An et al. [192] identify resistin as a mediator of insulin resistance and pro-inflammatory activity.

These studies converge on a critical insight: adipokine secretion, signalling, and metabolic impact are subject to epigenetic regulation. Consequently, dietary exposures, including bioactive compounds present in functional foods, may modulate adipokine profiles during adolescence, thereby shaping long-term metabolic trajectories in ways that are only beginning to be elucidated.

Thus, the evidence indicates that adipose-derived hormones such as leptin, adiponectin, and resistin constitute a highly responsive endocrine network. Their secretion patterns and metabolic actions are closely linked to nutritional status and may be modified through epigenetic mechanisms. Adolescence, therefore, represents a particularly sensitive window during which functional foods and their bioactive components may influence adipokine signalling and, ultimately, long-term metabolic health.

### 5.3. Adipokines and Epigenetically Regulated Signalling Pathways

Experimental evidence indicates that puberty represents a period of pronounced neuroendocrine plasticity. During this time, metabolic signals, environmental influences, and epigenetic processes converge to shape the long-term architecture of the hypothalamic–pituitary–gonadal (HPG) axis, as demonstrated in the study [201]. A growing body of research further suggests that obesity-related hormonal signals, particularly leptin, play a central role in this developmental window. As emphasised by Reinehr and Roth [202], leptin functions both as a metabolic messenger and as a permissive signal for pubertal initiation through its interaction with the kisspeptin system. This dual role implies that excess adiposity may accelerate or disrupt the timing of puberty. Supporting this concept, Huang et al. [203] show that leptin, nutritional status, and adipose-derived factors can modulate the tempo of pubertal development via intertwined endocrine and epigenetic pathways.

Environmental influences further complicate this relationship. Lee et al. [204] demonstrate that endocrine-disrupting chemicals (EDCs) can interfere with pubertal development by acting either directly on adipose tissue or centrally on the HPG axis. Early-life exposure to EDCs may induce persistent epigenetic alterations. Systematic evidence synthesised by Uldbjerg et al. [205] confirms that prenatal and postnatal exposure to EDCs is associated with shifts in pubertal timing in both girls and boys, although the magnitude and direction of these effects vary depending on the developmental window. Similar concerns have been raised regarding male pubertal maturation, where EDCs may alter normative trajectories through androgen-modulating and epigenetic mechanisms [206]. Reviews by Papadimitriou and Papadimitriou [207] and López-Rodríguez et al. [208] further highlight that EDCs can influence pubertal processes via chromatin remodelling, DNA methylation, and altered transcriptional regulation within hypothalamic circuits.

Obesity itself remains a potent modifier of these pathways. Wagner et al. [209] and Huang and Roth [210] note that excessive adiposity can disrupt sexual maturation by altering leptin signalling, aromatase activity, insulin sensitivity, and inflammatory pathways. Such alterations may leave durable epigenetic marks during this sensitive developmental period. Collectively, these findings indicate that puberty is not regulated solely by intrinsic hormonal mechanisms but is substantially shaped by metabolic, environmental, and epigenetic influences. Leptin signalling and EDC-related pathways, therefore, emerge as integral components of any comprehensive framework addressing pubertal timing and developmental programming.

Cytokines such as adiponectin and leptin, as highlighted by Tragomalou et al. [195], play a central role in the pathogenesis of metabolic syndrome. Their work is particularly valuable because it frames these adipocyte-derived hormones not merely as biomarkers but as active mediators of metabolic dysfunction. Ghadge and Khaire [194] and Ghadge et al. [196] further expand this perspective by demonstrating that leptin and adiponectin are closely linked to energy expenditure, lipid and glucose metabolism, and inflammatory regulation, thereby establishing these adipocytokines as crucial regulators of metabolic homeostasis. Alterations in their circulating levels have been consistently associated with an increased risk of obesity, insulin resistance, T2DM, and cardiovascular disease, underscoring the importance of maintaining adipokine balance for metabolic health [194].

Leptin initiates one of the principal adipokine-mediated signalling cascades by binding to its long-form receptor (Ob-Rb), which activates Janus kinase 2 (JAK2) and subsequently phosphorylates STAT3. This enables STAT3 nuclear translocation and transcriptional regulation of genes governing appetite, energy expenditure, and inflammatory tone. In parallel, adiponectin engages a distinct yet metabolically complementary pathway by activating AMP-activated protein kinase (AMPK) and PPARα, thereby enhancing fatty acid oxidation, improving insulin sensitivity, and suppressing hepatic gluconeogenesis, mechanisms that collectively counteract obesity-associated metabolic disturbances.

In contrast to these protective adipokines, resistin exerts deleterious effects by activating TLR4, which triggers downstream NF-κB and MAPK signalling pathways, promoting inflammation, insulin resistance, and endothelial dysfunction. These inflammatory responses are further amplified through NF-κB-dependent chromatin remodelling, which enhances transcription of pro-inflammatory genes. Additionally, specific microRNAs, including miR-199a and miR-155, fine-tune resistin-induced inflammatory cascades, illustrating the close integration between adipokine signalling and epigenetic regulation.

**Figure 4:**
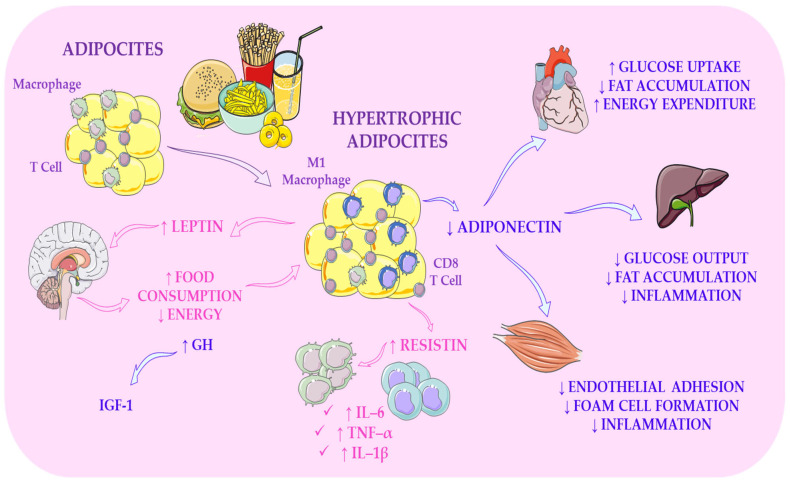
Adipokines as key endocrine regulators of metabolic homeostasis. Adipocytes function as active endocrine cells, secreting adipokines that integrate nutritional cues with systemic metabolic regulation. Leptin reflects energy stores and modulates hypothalamic signalling; adiponectin exerts anti-inflammatory and cardioprotective effects; whereas resistin promotes insulin resistance and pro-inflammatory activity. Adipokine signalling is highly sensitive to dietary composition and appears particularly modifiable during adolescence, a developmental period characterised by heightened metabolic and epigenetic plasticity. Image provided by Servier Medical Art (https://smart.servier.com/) (accessed on 5 January 2026), licensed under CC BY 4.0 (https://creativecommons.org/licenses/by/4.0/, accessed on 5 January 2026). Abbreviations: CD8—cluster of differentiation 8; GH—growth hormone; IGF-1—insulin-like growth factor 1; IL-1β—interleukin 1 beta; IL-6—interleukin 6; M1—classically activated macrophages (M1 macrophages); T cells—T lymphocytes; TNF-α—tumor necrosis factor alpha. ↑—increase in activity/level/expression; ↓—decrease in activity/level/expression.

Figure 4 highlights adipokines as pivotal endocrine regulators of metabolic homeostasis, illustrating how their coordinated actions influence energy balance, insulin sensitivity, inflammatory tone, and the overall metabolic phenotype.

Adding another layer of complexity, adipose tissue releases exosomal microRNAs that function as endocrine messengers capable of reprogramming gene expression in distant organs such as the liver and brain. This extends the metabolic influence of adipose tissue far beyond its anatomical boundaries. These adipokine-driven pathways converge on the insulin signalling cascade involving insulin receptor substrate-1 (IRS-1), phosphoinositide 3-kinase (PI3K), and Akt. Leptin and adiponectin enhance insulin sensitivity by promoting IRS-1 phosphorylation, PI3K activation, and Akt-mediated GLUT4 translocation. In contrast, resistin and other pro-inflammatory adipokines inhibit these processes, thereby impairing glucose uptake and reducing metabolic flexibility. Collectively, these interconnected molecular pathways illustrate how adipokines regulate metabolic homeostasis through receptor-mediated signalling, inflammatory modulation, and epigenetic mechanisms, underscoring their pivotal role in shaping metabolic health during adolescence.

Yanai and Yoshida [197] provide compelling evidence of the mechanisms underlying adiponectin’s anti-inflammatory and anti-atherogenic effects, including inhibition of TNF-α and IL-6, as well as reductions in LDL cholesterol and triglyceride concentrations. Tragomalou et al. [195] further demonstrate that higher adiponectin levels are associated with a reduced risk of myocardial infarction and improved post-ischaemic cardiac recovery. Together, these findings position adiponectin as a protective metabolic signal. However, Javier et al. [198] report no significant differences in adiponectin levels between adolescents with and without metabolic syndrome. This discrepancy may reflect differences in developmental stage, sample heterogeneity, or methodological variation, an important reminder that adolescent physiology cannot be directly extrapolated from adult data.

Pro-inflammatory adipokines such as TNF-α and IL-6 initiate intracellular signalling cascades that culminate in activation of the NF-κB, JNK, and p38 MAPK pathways. Collectively, these signalling axes promote insulin resistance, disrupt lipid handling, and exacerbate systemic metabolic dysregulation. Importantly, inflammatory signalling does not operate in isolation but is tightly integrated with epigenetic mechanisms that reshape the transcriptional landscape of metabolic tissues.

Chronic inflammation has been shown to induce global DNA hypomethylation while simultaneously driving site-specific hypermethylation of genes involved in glucose and lipid metabolism, thereby reinforcing metabolic dysfunction through sustained transcriptional reprogramming. Histone deacetylases (HDACs) further modulate chromatin accessibility at inflammatory gene promoters, enhancing or repressing transcription depending on cellular context. In parallel, specific microRNAs, most notably miR-146a and miR-21, fine-tune inflammatory responses by targeting key components of the NF-κB and MAPK pathways. Together, these findings support the concept that inflammation represents a transcriptionally encoded and epigenetically stabilised phenotype, rather than merely a transient biochemical state.

When considered alongside the PPARγ pathway, the master regulator of adipocyte differentiation, this inflammatory-epigenetic interface becomes even more consequential. PPARγ governs adipogenesis, lipid storage, adipokine secretion, and insulin sensitivity, positioning it as a central determinant of adipose tissue function. Its activity is highly dependent on epigenetic regulation: hypermethylation of the PPARG promoter reduces the differentiation capacity of precursor cells, whereas histone acetylation enhances PPARγ transcriptional activity and supports healthy adipocyte maturation. Moreover, microRNAs such as miR-27a and miR-130 suppress PPARγ expression, inhibiting adipogenesis and contributing to the expansion of dysfunctional adipose tissue. Disruption of these regulatory layers, whether driven by chronic inflammation, nutrient excess, or hormonal imbalance, leads to an adipose phenotype characterised by impaired lipid storage, aberrant adipokine secretion, and heightened metabolic risk.

These pathways demonstrate that adipokines act through an intricately coordinated network of molecular cascades, including JAK/STAT, AMPK, NF-κB, PI3K/Akt, and PPARγ signalling. The activity of these pathways is profoundly influenced by epigenetic mechanisms such as DNA methylation, histone modifications, and microRNA regulation. Given that these regulatory systems remain highly plastic during adolescence, dietary bioactive compounds present in functional foods may have the capacity to reprogram adipokine signalling and thereby influence long-term metabolic health trajectories.

**Figure 5:**
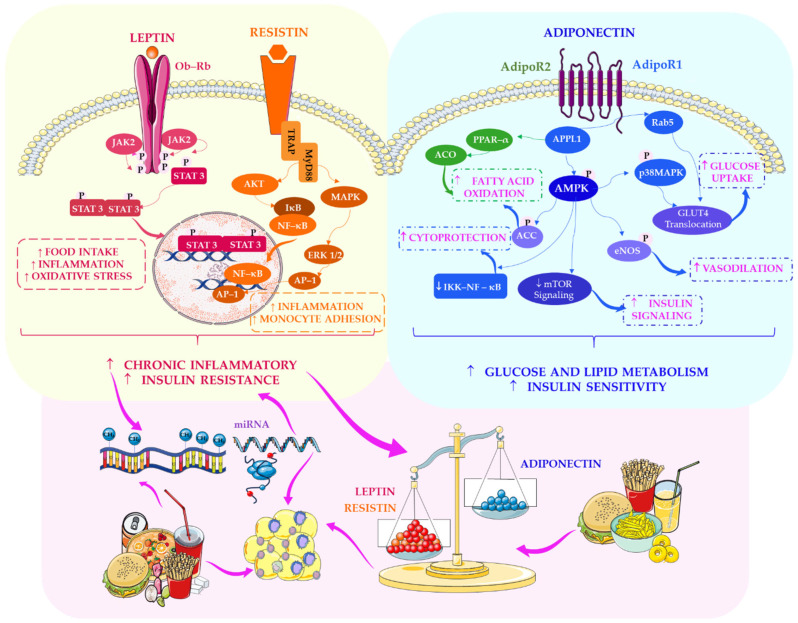
Integrated molecular pathways of adipokines: adiponectin, leptin, and resistin in the regulation of metabolism and inflammation Adipokines orchestrate metabolic homeostasis and inflammatory responses through an interconnected network of signalling pathways, whose activity is strongly modulated by epigenetic mechanisms. Adiponectin engages the AdipoR1/R2-APPL1-AMPK cascade, promoting fatty acid β-oxidation via PPARα/ACO, enhancing endothelial function through eNOS activation, and inhibiting NF-κB-mediated pro-inflammatory signalling. Leptin, which exhibits both pro-inflammatory and anabolic effects, signals through the ObRb receptor and JAK2 kinase, leading to STAT3 activation and downstream pathways that stimulate inflammatory responses. In contrast, resistin acts via TLR4, directly activating NF-κB and increasing the expression of pro-inflammatory cytokines. Image provided by Servier Medical Art (https://smart.servier.com/) (accessed on 5 January 2026), licensed under CC BY 4.0 (https://creativecommons.org/licenses/by/4.0/, accessed on 5 January 2026). Abbreviations: ACC—acetyl-CoA carboxylase; ACO—acyl-CoA oxidase; AdipoR1—adiponectin receptor 1; AdipoR2—adiponectin receptor 2; AKT—protein kinase B (Akt); AMPK—AMP-activated protein kinase; AP-1—activator protein 1; APPL1—adaptor protein, phosphotyrosine interacting with PH domain and leucine zipper 1; ERK1/2—extracellular signal-regulated kinases 1 and 2; eNOS—endothelial nitric oxide synthase; GLUT4—glucose transporter type 4; IKK—IκB kinase; IκB—inhibitor of nuclear factor kappa-B beta; JAK2—janus kinase 2; MAPK—mitogen-activated protein kinase; mTOR—mechanistic target of rapamycin; MYD88—myeloid differentiation primary response 88; NF-κB—nuclear factor kappa-light-chain-enhancer of activated B cells; Ob-Rb—long isoform of the leptin receptor; p38 MAPK—p38 mitogen-activated protein kinase; PPARα—peroxisome proliferator-activated receptor alpha; Rab5—ras-related protein Rab-5; STAT3—signal transducer and activator of transcription 3; TRAP—TNF receptor-associated protein; TLR4—Toll-like receptor 4. ↑—increase in activity/level/expression; ↓—decrease in activity/level/expression.

This interpretation aligns with broader evidence linking hyperleptinaemia and leptin resistance to obesity and insulin resistance. Francisco et al. [199] and Ghadge and Khaire [194] suggest that leptin concentrations exceeding 13.4 ng/dL may serve as an early indicator of metabolic syndrome risk in prepubertal children, underscoring both the diagnostic and mechanistic relevance of leptin signalling in paediatric populations. As illustrated in Figure 5, adiponectin, leptin, and resistin operate within an integrated molecular network that coordinates metabolic regulation and inflammatory signalling. The activity of this network is further modulated by epigenetic mechanisms, collectively shaping systemic metabolic homeostasis and immune function.

Adipocytes are now widely recognised as metabolically active cells that secrete more than a dozen hormones influencing appetite, satiety, and whole-body energy metabolism. While leptin, the first adipocyte-derived hormone to be identified, suppresses appetite, and genetic absence of leptin results in severe obesity, Saklayen [200] notes that adiponectin exerts the opposite metabolic effect, thereby highlighting the functional diversity of adipokines. Resistin, as described by An et al. [192], mediates insulin resistance and is associated with pro-inflammatory activity and elevated cardiometabolic risk. This further illustrates the multifaceted endocrine role of adipose tissue.

An important advancement in this field comes from Thomou et al. [211], who demonstrated that microRNAs derived from adipose tissue in the form of exosomes can regulate gene expression in distant organs such as the liver, effectively functioning as adipokines themselves. This concept was reinforced by Wang et al. [212], who showed that exosomes derived from the adipose tissue of mice fed a high-fat diet caused synaptic damage in the hippocampus and cortex, thereby impairing cognitive function. In this process, miR-9-3p was identified as a key player. An et al. [192] interpret these findings as a potential mechanistic explanation for cognitive impairment associated with metabolic syndrome, emphasising the wide-reaching systemic consequences of adipose tissue dysfunction.

Dysfunctional adipose tissue in obesity predisposes individuals to metabolic disease through multiple, mutually reinforcing mechanisms, including chronic low-grade inflammation, elevated circulating free fatty acids, ectopic lipid accumulation, and profound alterations in the adipose secretory profile [192]. These disturbances are particularly consequential during adolescence, a period of rapid hormonal and metabolic remodelling that amplifies their physiological impact. As the adolescent endocrine milieu is highly malleable, disruptions in adipose-derived signalling at this stage can have disproportionate and long-lasting effects on metabolic programming, thereby increasing the risk of insulin resistance, dyslipidaemia, and cardiometabolic disorders in adulthood.

Against this backdrop of heightened developmental vulnerability, functional foods emerge as biologically plausible and mechanistically grounded interventions. Their bioactive components can modulate epigenetic processes, including DNA methylation, histone modifications, and microRNA regulation, which govern the expression of genes essential for metabolism, insulin sensitivity, and lipid homeostasis. During adolescence, when endocrine and metabolic systems undergo significant restructuring, dietary patterns rich in functional food components may serve as an epigenetic strategy to counteract the adverse metabolic imprinting associated with adipose dysfunction, ultimately reducing the risk of metabolic and hormonal disorders later in life.

Thus, adipokines such as leptin, adiponectin, and resistin, together with adipose-derived microRNAs, constitute a complex endocrine network whose signalling is tightly linked to metabolic risk and highly responsive to dietary modulation. This makes adolescence a critical window during which functional foods can exert long-lasting epigenetic and metabolic effects.

### 5.4. Fatty Acids as Epigenetic Regulators of Metabolic Pathways

Fatty acids (FAs) are increasingly recognised as key dietary modulators of the epigenome, with profound implications for the prevention of non-communicable chronic diseases (NCCDs). Tremblay et al. [213] emphasise that omega-3 polyunsaturated fatty acids (n-3 PUFAs) and short-chain fatty acids (SCFAs) exert particularly strong protective effects, largely via epigenetic mechanisms that regulate gene expression. Other classes of fatty acids, including n-6 PUFAs, MUFAs, SFAs, and TFAs, also influence epigenetic programming, particularly through alterations in DNA methylation [214]. PUFAs can modulate DNA methylation, histone modifications, and microRNA expression, thereby shaping metabolic gene activity. While n-3 PUFAs (e.g., EPA and DHA) and certain MUFAs are associated with reduced risk of obesity, T2DM, and fatty liver disease, n-6 PUFAs, SFAs, and TFAs tend to promote these metabolic disturbances [140].

Recent advances have highlighted valeric acid (VA) as a physiologically relevant, gut-derived SCFA with potent epigenetic activity. According to Paciolla et al. [215], VA acts as a selective inhibitor of class I histone deacetylases, particularly HDAC3, positioning it as a key microbial metabolite capable of modulating neuroinflammatory pathways within the gut–brain axis. In addition to its epigenetic effects, VA exerts GABAergic neuroprotective actions, distinguishing it from classical SCFAs such as acetate, propionate, and butyrate. Notably, VA is a safer endogenous analogue of valproic acid, providing targeted HDAC inhibition without the systemic toxicity associated with pharmacological HDAC inhibitors. These properties establish VA as a promising candidate for linking gut dysbiosis, neuroinflammation, and the epigenetic programming of neuroendocrine circuits, particularly during adolescence, when significant remodelling occurs in both the microbiota and the central nervous system (CNS).

Any meaningful discussion of lipid biology in adolescence must also consider the omega-6/omega-3 ratio, an increasingly recognised metabolic and neurobiological pivot point. As Tureck et al. [216] compellingly demonstrate using data from a large Brazilian cohort, the average ratio of omega-6 to omega-3 fatty acids in contemporary adolescent diets is 7.93:1. This nutritional landscape mirrors global dietary trends and may predispose young people to pro-inflammatory metabolic profiles. In an earlier systematic review [217], the authors further emphasise that the heterogeneity of omega-6 intake across studies complicates the interpretation of omega-3 effects on components of metabolic syndrome. They conclude that the ratio itself, rather than isolated fatty acids, may be the more biologically meaningful metric.

Clinical evidence from paediatric populations supports this view. Trebatická et al. [218] demonstrated that targeted omega-3 supplementation reduced depressive symptoms in children and adolescents, while also markedly lowering the omega-6 to omega-3 ratio from 24.2:1 to 7.6:1. This finding suggests a direct link between lipid balance and neuropsychiatric outcomes. In a complementary randomised trial, Katrenčíková et al. [219] reported that omega-3 fatty acids increased anti-atherogenic large HDL subfractions and decreased pro-atherogenic small HDL particles, an effect not observed with omega-6 supplementation. These results highlight the functional consequences of restoring lipid equilibrium.

Experimental studies provide a more precise understanding of the underlying mechanisms. Frajerman et al. [220] showed that lifelong exposure to omega-6-dominant diets alters anxiety, impulsivity, and cognitive flexibility in rodents, whereas a balanced omega-6/omega-3 intake normalises these behavioural phenotypes. Similarly, Soleimanzad et al. [221] demonstrated that an imbalanced omega-6/omega-3 ratio in a Western diet impairs cerebrovascular functional hyperemia in adolescent mice, an effect fully reversible through DHA-based rebalancing of the ratio. Observational human data are consistent with these findings: da Rocha and Kac [222] reported that pregnant women with an omega-6/omega-3 ratio exceeding 9:1 had significantly higher rates of postpartum depression, suggesting that excessive omega-6 intake may influence inflammatory and neuroendocrine pathways throughout life.

These findings converge on a clear conclusion: the omega-6 and omega-3 ratio is a biologically active determinant of inflammatory tone, metabolic resilience, and neurobehavioral trajectories in adolescents, not merely a nutritional descriptor. It is therefore essential to include it in any comprehensive discussion of lipid-related mechanisms in young people.

At the molecular level, n-3 PUFAs enhance DNMT activity and cooperate with the methyl-CpG-binding protein MeCP2, thereby activating nuclear receptors such as PPARγ and reducing the expression of pro-inflammatory cytokines [140,223,224]. Short-chain fatty acids (SCFAs), particularly butyrate, act as potent HDAC inhibitors, directly modifying chromatin accessibility and gene transcription. As microbial fermentation products, SCFAs form a central component of the diet–microbiota–epigenetics axis [224]. Other dietary compounds also modulate epigenetic enzymes: sulforaphane (SFN), found in cruciferous vegetables, inhibits nuclear HDACs, rapidly altering histone–DNA interactions and gene expression [225], while betaine stabilises DNA methylation at the promoters of cholesterol metabolism genes, such as HMGCR and CYP7A1, thereby protecting against hepatic cholesterol accumulation [189]. Fermentable fibre further enhances SCFA production, influencing the activity of epigenetic enzymes and metabolic gene expression [226]. These mechanisms are particularly relevant during adolescence, when the demand for amino acids, calcium, and vitamin D is high due to rapid tissue expansion and epigenetic plasticity is exceptionally pronounced [1].

### 5.5. Polyphenols as Multifunctional Epigenetic Modulators

Polyphenols are chemically diverse compounds, including flavonoids (such as flavanols, anthocyanins, and isoflavones) and non-flavonoids (including phenolic acids, stilbenes, and lignans) [212]. They are abundant in fruits, vegetables, grains, tea, and wine [212,227]. As a major class of phytochemicals, polyphenols mediate dynamic interactions between the genome and the environment [121], earning recognition as multifunctional epigenetic modulators. Recent evidence indicates that polyphenols may reverse adverse epigenetic changes associated with obesity, metabolic dysfunction, cardiovascular disease, neurodegeneration, and cancer [20]. Their molecular actions include modulation of DNA methylation, histone modifications, and microRNA expression [228]. Many polyphenols act as histone deacetylase (HDAC) inhibitors, altering histone acetylation and methylation patterns and regulating genes involved in inflammation, oxidative stress, apoptosis, and cell-cycle control. The distribution and conjugation patterns of hydroxyl groups largely determine their epigenetic specificity [229].

Dietary intake varies by dietary pattern: Mediterranean diets provide approximately 700 mg/day of polyphenols [230], whereas Western diets range from 300 to 1100 mg/day [227]. Beyond their antioxidant capacity, which reduces systemic inflammation, a key driver of obesity and MetS [212,227], polyphenols also modulate immune responses and glucose-lipid metabolism [231,232].

Specific polyphenols exhibit distinct molecular actions. Strawberry polyphenols demonstrate hypoglycaemic and hypolipidaemic effects in diabetic rats [233]. Epigallocatechin gallate (EGCG), the predominant catechin in green tea, exerts antioxidant, anti-inflammatory, cardioprotective, and anticancer effects [187], and increases Ucp1 expression in brown adipose tissue [234]. Chlorogenic acid activates AMPK, inhibits HMG-CoA reductase, and enhances carnitine palmitoyltransferase activity [70,235,236]. Quercetin regulates lipogenic gene expression [237], whereas phlorizin inhibits SGLT1/2, reducing glucose absorption and improving postprandial glycaemia [238,239]. Curcumin modulates genes associated with MetS via PPARγ activation [240] and improves lipid profiles, glycaemia, and inflammation in clinical trials [241,242,243,244]. Berberine exhibits hypoglycaemic, hypolipidaemic, anti-inflammatory, and microbiota-modulating effects [212,245,246], and improves metabolic and hormonal profiles in polycystic ovary syndrome (PCOS) [247,248].

### 5.6. Diet–Microbiota–Epigenome Interactions

Adolescence is a period of exceptional physiological plasticity, during which the gut microbiome emerges as a critical regulator of metabolic maturation. Dynamic hormonal shifts, rapid somatic growth, and increased energy requirements intensify the interactions between microbial metabolites and host tissues. The gut microbiome plays a central role in maintaining metabolic homeostasis, controlling biochemical pathways that influence energy balance, glucose and lipid metabolism, and bile acid turnover [249]. Through both metabolic activity and molecular signalling, the microbiota exerts profound effects on physical health and neuropsychological function, serving as a bidirectional interface between the gut and the brain [250].

Evidence increasingly indicates that the gut microbiota undergoes characteristic maturation during puberty, closely linked to rising sex steroid levels and associated metabolic changes. Calcaterra et al. [251] highlight that puberty is marked by the emergence of sex-specific microbial signatures, reflecting the bidirectional communication between the gut microbiome and the endocrine system. Their review also reports that alterations in microbial composition have been observed in girls with central precocious puberty, suggesting that the sex hormone-gut microbiome axis may influence the timing of pubertal onset. Expanding on this concept, Yue and Zhang [252] describe the pathways through which microbiota may modulate pubertal regulation. These include the modulation of GnRH, LH, and FSH secretion; the production of neuroactive metabolites by the microbiota; and interactions with metabolic pathways linked to obesity, a well-established risk factor for early puberty.

Empirical evidence supports these mechanistic insights. Wang et al. [253] demonstrated that girls with obesity-related precocious puberty exhibit distinct microbial profiles, with significant differences in α- and β-diversity. Specific taxa, including *Bifidobacterium*, *Anaerostipes*, *Bacteroides*, and *Ruminococcus gnavus*, emerged as biomarkers. Notably, *Anaerostipes* abundance was negatively correlated with BMI, bone age, LH, FSH, and estradiol, reinforcing the link between microbial composition and metabolic and endocrine maturation. These findings align with earlier evidence summarised by Indiani et al. [254], who reported that changes in the *Firmicutes* to *Bacteroidetes* ratio, specifically, an increase in *Firmicutes* and a decrease in *Bacteroidetes*, are strongly associated with childhood obesity, thereby connecting microbial dysbiosis to a key contributor to precocious puberty.

Additional mechanistic layers have been revealed through studies of oxidative stress, neuroimmune signalling, and the gut–brain axis. Research [255,256,257,258] converges on the idea that gut microbiota composition modulates responses to oxidative stress, neuroimmune pathways, vagal signalling, and metabolic resilience—all of which are relevant to the timing of puberty. These studies demonstrate that microbial metabolites and antioxidant pathways influence neuroendocrine circuits, including those regulating reproductive maturation. Moreover, as discussed by Tkaczenko and Kurhaluk [259], functional foods and antioxidant-rich diets can modulate Nrf2-related pathways, indirectly affecting microbiota-dependent endocrine regulation.

These studies support a coherent model in which puberty is not solely hormonally orchestrated but also microbially co-regulated. In this framework, sex steroids shape the microbiota, which in turn influences neuroendocrine maturation. This reciprocal relationship provides a compelling explanation for both normal pubertal development and the rising prevalence of precocious puberty, particularly in the context of obesity and environmental stressors. The literature strongly supports considering the gut microbiota as a key biological component in models of pubertal timing and metabolic risk.

Comparative studies consistently demonstrate that individuals with obesity or MetS exhibit a markedly different gut microbial composition compared to lean individuals, highlighting the microbiome’s contribution to metabolic phenotypes [260]. Diet is therefore one of the most powerful modulators of microbial ecology, shaping both the taxonomic structure and functional capacity of the gut ecosystem, as shown by Wang et al. [249]. During adolescence, microbial products such as short-chain fatty acids (SCFAs) activate key metabolic pathways, including AMPK, GPR41/43, PPARγ, and mTOR, modulating insulin sensitivity, adipogenesis, mitochondrial biogenesis, and lipid oxidation. Simultaneously, microbial regulation of bile acid pools influences FXR-FGF19 and TGR5 signalling, which are essential for glucose homeostasis and energy expenditure. As the adolescent immune system is still developing, microbial-associated molecular patterns (MAMPs) can strongly regulate TLR-NF-κB, NOD1/2, and MAPK pathways, determining the balance between physiological inflammation and pathological metabolic stress. These mechanisms position the microbiome as a critical determinant of long-term metabolic programming during adolescence.

It is well established that indigestible dietary components, such as fibre and certain plant-derived sugars, serve as substrates for microbial fermentation, producing metabolites that act as systemic signalling molecules. The microbiome also participates in the biotransformation of bile acids, vitamins, dietary bioactives, and host-derived compounds, integrating nutritional signals with host metabolic pathways [261]. Microbial composition and function are further shaped by macronutrient distribution (fibre, fats, and proteins), the balance of plant-based versus high-fat foods, lifestyle factors, and early-life environmental exposures, while genetic and immunological predispositions modulate colonisation and metabolic output [262,263].

Certain microbial configurations enhance energy extraction from the diet, predisposing individuals to weight gain despite comparable caloric intake [261,264]. Microbial metabolites, particularly SCFAs, act on adipose tissue and the liver to regulate lipogenesis, lipid storage, and insulin sensitivity via GPR41/43 signalling, AMPK activation, and PPARγ modulation. The microbiome also influences bile acid metabolism, affecting FXR and TGR5 signalling, which are critical for lipid homeostasis and energy expenditure [265]. Dysbiosis disrupts these pathways, contributing to impaired glucose and lipid metabolism, mitochondrial dysfunction, and chronic low-grade inflammation—hallmarks of metabolic disease [266].

The gut microbiota, comprising trillions of microorganisms, plays a central role in metabolic regulation, as several studies have demonstrated [190]. Early dietary patterns can shape microbiome composition and contribute to long-term metabolic outcomes, including obesity and related disorders [267]. Short-chain fatty acids (SCFAs) produced by microbial fermentation act as epigenetic mediators, influencing HDAC activity and metabolic gene expression. Many polyphenols require microbial metabolism to become bioactive, and their microbial-derived metabolites often exhibit stronger biological activity than their dietary precursors.

A key physiological function of the microbiota is maintaining intestinal barrier integrity. By supporting tight junction proteins and producing SCFAs such as butyrate, the microbiota limits intestinal permeability (“leaky gut”) and prevents excessive translocation of inflammatory molecules into the bloodstream [266]. Dysbiosis, however, disrupts barrier function, allowing MAMPs, including lipopolysaccharide (LPS) and peptidoglycan, to enter circulation. These MAMPs activate innate immune receptors and promote systemic inflammation [268]. Diets high in saturated fats and sucrose favour the growth of Gram-negative bacteria, which release peptidoglycans recognised by the NOD1 receptor. Activation of NOD1 triggers downstream NF-κB and MAPK signalling cascades, leading to insulin resistance and hyperglycemia in obesity [269]. Moreover, NOD1 expression itself is modulated by the microbiota, amplifying inflammatory responsiveness [270,271].

Recent findings indicate that inhibition of the TLR4/TRIF-MyD88-NF-κB axis can prevent diet-induced insulin resistance, highlighting a mechanistic link between high-fat diets, microbial MAMPs, and metabolic inflammation [272]. Chronic low-grade inflammation associated with Western dietary patterns is therefore closely tied to microbiota-derived molecular signals that influence host metabolic regulation [273].

Both short- and long-term dietary interventions can rapidly remodel the microbiome [274]. Diet shapes microbial composition and functional outputs, including metabolite production, barrier integrity, and immune modulation [275]. Undigested dietary proteins (10–30%) and fatty acids (~5%) serve as substrates for microbial metabolism, yielding either beneficial SCFAs or potentially harmful metabolites from proteolysis and lipolysis, which influence gut health and systemic immunity [276].

Phytoestrogens illustrate the interplay between diet, microbiota, and metabolic regulation particularly well. Found in soy products, fruits, vegetables, and dairy [277,278,279], they can act as endocrine modulators, with potential disruptive effects on hormonal signalling [280,281]. Their biological activity depends not only on the ingested free forms but also on gut-derived metabolites, which can interfere with endogenous estrogen signalling [282]. Only 5–10% of ingested phytoestrogens reach the small intestine for direct absorption, while the majority undergo extensive metabolism in the liver and intestines. This metabolism produces compounds such as S-equol, O-DMA, enterolignans, and stilbene derivatives, which often exhibit greater bioactivity than their precursors [283,284,285]. These processes underscore the critical role of the microbiota as a mediator linking diet, epigenetic regulation, and metabolic health.

Diets rich in fibre-including fruits, vegetables, whole grains, legumes, nuts, and fish-combined with reduced intake of red meat, refined sugars, and ultra-processed foods, create an environment that supports microbial diversity and metabolic resilience [286,287]. Plant-based and Mediterranean dietary patterns enhance SCFA production and reduce trimethylamine N-oxide (TMAO), a metabolite associated with cardiometabolic risk. These diets promote microbial diversity, which is strongly associated with a lower incidence of non-communicable diseases [288]. Increased abundance of *Faecalibacterium*, *Prevotella*, and *Bacteroides* has been linked to improved glycemic control, reduced adiposity, enhanced bowel function, lower inflammation, and decreased hospitalisation risk [289]. Diets rich in prebiotics, fermented foods, and plant-derived bioactives, including polyphenols and flavonoids, further enhance microbial stability and diversity [290].

The microbiome is now recognised as a dynamic, metabolically active organ whose composition, variability, and resilience influence the onset, progression, and treatment of numerous diseases [291]. Dysbiosis has been implicated in inflammatory bowel disease, obesity, type 2 diabetes, allergies, liver disease, metabolic disorders, and mental health conditions [291,292]. Microbiome profiling is increasingly used to evaluate metabolic risk, lipid imbalance, and susceptibility to obesity, reflecting the integrated effects of diet, lifestyle, and host physiology [293].

Table 4 summarises the main epigenetic mechanisms affected by bioactive dietary compounds, detailing the molecular pathways targeted and the resulting metabolic effects.

Thus, the evidence indicates that the adolescent microbiome functions as a metabolic and immunological “switchboard”, integrating dietary cues, hormonal signals, and environmental influences to produce epigenetically mediated physiological outcomes. By modulating pathways such as NF-κB, TLR4/TRIF-MyD88, FXR/TGR5, AMPK, and PPARγ, the microbiota regulates both immediate metabolic flexibility and long-term molecular signatures that shape susceptibility to obesity, insulin resistance, NAFLD, and cardiometabolic disease in adulthood. Accordingly, the plasticity of the microbiome during adolescence represents a double-edged sword: it confers vulnerability to dysbiosis induced by poor diet and lifestyle, yet also presents an opportunity for targeted nutritional and lifestyle interventions capable of recalibrating host metabolism at a molecular and epigenetic level.

## 6. Developmental and Environmental Modifiers of Epigenetic Metabolism

Metabolic diseases represent a significant global public health challenge [302]. Their development is influenced by complex interactions between environmental factors, lifestyle, and epigenetic regulation. Adolescence, characterised by rapid growth and heightened epigenetic sensitivity, is a particularly critical window during which nutrient availability and environmental exposures can have long-lasting effects on metabolic health. Glucose homeostasis is tightly regulated by insulin, glucagon, adrenaline, noradrenaline, cortisol, and growth hormone, and disruptions in these pathways can result in hypoglycemia [303]. These findings underscore that adolescence is a pivotal period during which diet, the microbiota, and environmental factors converge to shape the epigenome and long-term metabolic trajectory.

Environmental stressors can also influence epigenetic regulation. For example, acute exposure to PM_2.5_ alters DNA methylation patterns associated with inflammation and oxidative stress. In healthy adults, PM_2.5_ exposure reduces mitochondrial DNA content by 11.1%, but B-vitamin supplementation can prevent these epigenetic disturbances [131]. Dietary interventions further illustrate the potential to counteract environmental and metabolic stress. Omega-3 fatty acids, particularly EPA and DHA from fish oil, stimulate thermogenesis in brown adipose tissue, upregulating thermogenic markers such as Adrb3, Pgc1a, and Ucp1, thereby preventing diet-induced obesity [234,304]. Capsaicin, acting through TRPV1 and PPARα, mitigates metabolic disturbances by increasing adiponectin and its receptor expression [200].

Thus, functional foods can meaningfully complement the adolescent diet by supporting immunity, metabolic regulation, and the prevention of lifestyle-related diseases. However, their benefits should be considered adjunctive rather than a replacement for healthy dietary habits. Overreliance on these products may foster the false impression that they can compensate for poor nutrition or an unhealthy lifestyle. Accordingly, strengthening nutritional literacy among young people is crucial, particularly in an environment saturated with persuasive marketing. Long-term, well-designed studies remain necessary to clarify the effects of functional foods on metabolic, neuroendocrine, and immunological health in adolescents and to establish evidence-based guidelines for their safe and rational use during this critical developmental period.

### 6.1. Limitations

Current understanding of how functional foods modulate epigenetic mechanisms during adolescence is constrained by several factors. First, mechanistic studies specifically targeting this age group are limited, and much of the evidence relies on extrapolation from adult or animal models. Research is further complicated by substantial heterogeneity in study designs, dietary exposures, epigenetic endpoints, and analytical methods, which hampers cross-study comparisons and weakens causal inference. Additionally, the majority of existing evidence stems from short-term interventions and observational studies, providing limited insight into long-term epigenetic trajectories and their sustained impact on metabolic health during the transition from adolescence to adulthood.

### 6.2. Future Research

To advance this field, integrated multi-omics approaches are needed to delineate how specific bioactive components of functional foods modulate epigenetic pathways during adolescence, a period of heightened developmental plasticity. Longitudinal cohort studies and rigorously controlled intervention trials are required to determine the longevity, dose–response relationships, and clinical significance of these epigenetic modifications with respect to metabolic health trajectories. Future research should also address interindividual variability, including genetic background, microbiome composition, and lifestyle factors, to enable the development of personalised nutritional strategies targeting epigenetic mechanisms in young people.

## 7. Conclusions

Adolescence represents a particularly sensitive period during which dietary factors can exert disproportionate and long-lasting effects on metabolic health. Diets high in ultra-processed foods, excess sugars, and saturated fats disrupt metabolic homeostasis at an early stage, accelerating the development of obesity, insulin resistance, and related cardiometabolic disturbances. This vulnerability is amplified by the biological remodelling that occurs during puberty, when metabolic pathways, hormonal systems, and neural circuits regulating appetite and reward are still developing.

During this critical developmental window, long-term dietary habits and metabolic pathways are established. Diets rich in ultra-processed foods, simple sugars, and saturated fats disrupt insulin signalling, enhance lipogenesis via pathways such as SREBP-1c and ACC, and activate inflammatory cascades, including NF-κB. These perturbations accelerate the onset of obesity, IR, and other early-life metabolic disturbances.

Bioactive components of functional foods, including polyphenols, omega-3 fatty acids, and dietary fibre, modulate metabolic health through both direct biochemical actions and epigenetic mechanisms. These effects involve DNA methylation of key metabolic genes such as *PPARγ*, *FTO*, and *LEP*, as well as histone modifications and microRNA regulation (e.g., miR-122, miR-33, miR-375). By influencing these molecular pathways, diet can program gene expression patterns associated with inflammation, lipid metabolism, and insulin sensitivity.

The gut microbiota plays a central role in linking diet, epigenetic regulation, and metabolism. Diets rich in fibre and polyphenols promote the production of SCFAs by gut microbes. SCFAs act through GPR41/43 receptors and by inhibiting histone deacetylases, supporting anti-inflammatory effects and insulin sensitisation. In contrast, Western-style diets disrupt microbial composition, increase intestinal permeability, and activate TLR4-dependent inflammatory signalling, thereby exacerbating metabolic dysregulation.

Importantly, some diet-induced epigenetic alterations during adolescence, such as DNA methylation changes in metabolic genes or microRNA dysregulation, may persist into adulthood and potentially influence future generations via germline epigenetic programming. Accordingly, improving diet quality and incorporating functional food components during adolescence can enhance current metabolic health and reduce long-term and even intergenerational risk of metabolic disease through durable epigenetic effects.

## Figures and Tables

**Table 1:** Prevalence and clinical characteristics of selected metabolic disorders in paediatric and young adult populations.

Metabolic Disorder/Country	Prevalence/Indicator	Population/Age	Clinical Components	Source
Metabolic syndrome (India)	24%	Adolescents, 10–19 years	≥3 risk factors: obesity, dyslipidemia, hypertension, impaired glucose metabolism	[54]
Metabolic syndrome (Saudi Arabia)	7% overall; 30% in adolescents with BMI ≥ 95th percentile	Girls, 12–19 years	≥3 abnormal metabolic parameters	[55]
Insulin resistance (Turkey)	70.5%	Obese children and adolescents, 6–18 years	Elevated HOMA-IR; more common after puberty	[56]
Impaired glucose tolerance (Poland)	~16% among obese children	~10 years (exact range not reported)	Abnormal post-OGTT glucose response	[57]
Impaired fasting glucose (Poland)	~2.8% among obese children	~10 years (exact range not reported)	IFG diagnosed by OGTT criteria	[57]
Type 2 diabetes (Poland)	~3.45% among obese children	~10 years (exact range not reported)	T2DM diagnosed by OGTT	[57]
Type 2 diabetes—offspring of diabetic mothers (Denmark)	206 participants	Men, 30–31 years; O-GDM (n = 18), O-T1DM (n = 18), O-BP controls (n = 16)	Adipocyte hypertrophy (O-GDM); ↓ LEP promoter methylation, ↑ LEP expression and leptin secretion (O-GDM, O-T1DM); oxidative defects; ↑ lipolysis; ↓ fat-storage capacity	[58]
Obesity	234 participants; 15% with history of maltreatment	Children, 8–15 years	Increased BMI	[59]
NAFLD (Australia)	14.5% overall (17.4% girls; 11.8% boys)	Adolescents, 17 years (17.03 ± 0.26)	↑ BMI, ↑WC, ↑ leptin, ↑ HOMA-IR, ↑ fasting insulin, ↑ hsCRP, altered adiponectin, ↑ ALT, ↑ GGT	[21]

Abbreviations: ALT—alanine aminotransferase, BMI—body mass index, GGT—gamma-glutamyltransferase, GDM—gestational diabetes mellitus, HOMA-IR—Homeostatic Model Assessment of Insulin Resistance, hsCRP—high-sensitivity C-reactive protein, IFG—impaired fasting glucose, IGT—impaired glucose tolerance, IR—insulin resistance, LEP—leptin gene, MetS—metabolic syndrome, NAFLD—non-alcoholic fatty liver disease, OGTT—oral glucose tolerance test, O-BP—offspring from the background population (controls), O-GDM—offspring of mothers with gestational diabetes mellitus, O-T1DM—offspring of mothers with type 1 diabetes mellitus, T1DM—type 1 diabetes mellitus, T2DM—type 2 diabetes mellitus, WC—waist circumference. ↑—increase in activity/level/expression; ↓—decrease in activity/level/expression.

**Table 2:** Prevalence, nutritional and environmental determinants, epigenetic mechanisms, and clinical consequences of metabolic and cardiometabolic disorders in adolescents.

Disease/ Disorder	Prevalence (12–19 Years)	Nutritional and Environmental Factors	Epigenetic Mechanisms	Clinical Consequences	References
Obesity	20–22% of adolescents	Excess caloric intake, ultra-processed foods, sugar-sweetened beverages, physical inactivity	Hypermethylation of PPARγ, LEP, and ADIPOQ genes → impaired adipogenesis and appetite regulation	Insulin resistance, dyslipidemia, hypertension	[48,67]
Extreme obesity (BMI ≥ 160% of the 95th percentile)	1.13% (increase from 0.32% in 2008)	High intake of simple sugars and saturated fats	Persistent DNA methylation changes in HPA-axis and FTO genes; dysregulation of microRNAs (miR-122, miR-33)	NAFLD, prediabetes, metabolic syndrome	[67]
Metabolic syndrome (MetS)	3.3% overall; 29.2% among adolescents with obesity	High intake of trans fats, low dietary fiber, excess sodium	Altered methylation of INSR, IRS1; expression of miR-375 affecting insulin secretion	Dyslipidemia, hypertension, insulin resistance	[36,67]
Type 2 diabetes (T2DM)	0.46% in adolescents; increasing trend	Chronic hyperglycemia, high-fat diet	Epigenetic activation of TXNIP and methylation of Pdx1, leading to β-cell dysfunction	Nephropathy, retinopathy, cardiovascular disease	[68,69]
Non-alcoholic fatty liver disease (NAFLD)	10–20% of adolescents	Excess fructose intake, saturated fats, visceral obesity	Dysregulation of miR-34a and miR-122 → enhanced lipogenesis and hepatic inflammation	Fibrosis, steatohepatitis, risk of hepatocellular carcinoma	[70]
Dyslipidemia	13–15% of adolescents with obesity	High intake of trans fats and cholesterol	Methylation of APOA5, LPL, and CETP modulating lipid profile	Atherosclerosis and cardiovascular disease	[68,71]

Abbreviations: ADIPOQ—adiponectin gene, ALT—alanine aminotransferase, APOA5—apolipoprotein A5 gene, BMI—body mass index, CETP—cholesteryl ester transfer protein gene, CVD—cardiovascular disease, DNA—deoxyribonucleic acid, FTO—fat mass and obesity-associated gene, HFD—high-fat diet, HPA axis—hypothalamic–pituitary–adrenal axis, INSR—insulin receptor gene, IRS1—insulin receptor substrate 1 gene, LEP—leptin gene, LPL—lipoprotein lipase gene, miR/microRNA—microRNA, NAFLD—non-alcoholic fatty liver disease, PPARγ—peroxisome proliferator-activated receptor gamma, SETD2—SET domain-containing 2 gene, T2DM—type 2 diabetes mellitus, TXNIP—thioredoxin-interacting protein.

**Table 3:** Epigenetic modulators present in functional foods: bioactive molecules, targeted enzymes, and resulting metabolic adaptations.

Models	Bioactive Compound/Source	Epigenetic Mechanism	Epigenetically Mediated Biological Effects	References
In vitro (colon cancer cells)	Resveratrol	↑ H3K9/K14 acetylation at *p53*, *PTEN*	Activation of tumor-suppressor genes	[178,179]
In vitro (3T3-L1 adipocytes)	Resveratrol	Sirtuin activation → histone deacetylation	Modulation of metabolic gene expression	[180]
In vivo + in vitro	Gallic acid, sulforaphane, HDAC inhibitors	HDAC inhibition (class IIa, HDAC8)	Lower blood pressure; reduced HDAC activity	[181]
In vitro (intestinal cells/macrophages)	Polyphenols	DNMT/HDAC modulation	Reduced inflammation; altered metabolic genes	[182]
In vivo (ZF rats)	Dietary polyphenols	DNA methylation changes	Better glucose tolerance; lower lipids level	[183]
In vivo (metabolic models)	High-fiber, polyphenol-rich diet → SCFA	HDAC inhibition by SCFA	Improved glucose homeostasis; ↓ inflammation	[184]
In vitro (rat calvaria cells)	Buffalo casein peptides	Upregulation of osteogenic genes	↑ ALP, OCN, COL-1; ↑ mineralization	[185]
In vitro + in vivo (HFD mice, IF)	dietary pattern/strategy	Activation of p53 transcriptional pathway	↑ apoptotic/inflammatory gene expression	[186]
In vitro (human adipocytes)	EGCG	Likely histone/DNMT modulation	↓ oxidative stress; metabolic effects	[187]
In vivo (humans with obesity/IR)	Polyphenol- and fiber-rich diet	CpG/histone modification changes	Weight loss; improved glucose markers	[188]
In vivo (hens exposed to CORT)	Betaine	DNA methylation of *HMGCR*, *CYP7A1*	Normalized cholesterol metabolism	[189]
Animal + human models	High-fiber diet → SCFA	SCFA modulation of DNMT/HDAC, miRNA	Better glucose control; ↓ inflammation	[190]

Abbreviations: ALP—alkaline phosphatase, AMPK—AMP-activated protein kinase, C3H10T1/2—mouse embryonic fibroblast cell line, COL-1—collagen type I, CpG—cytosine-phosphate-guanine dinucleotide, CORT—corticosterone, CYP7A1—cholesterol 7α-hydroxylase, DNMT—DNA methyltransferase, EGCG—epigallocatechin gallate, FGF19—fibroblast growth factor 19, FXR—farnesoid x receptor, H3K9/K14—histone h3 lysine 9/lysine 14, HDAC—histone deacetylase, HFD—high-fat diet, HMGCR—3-hydroxy-3-methylglutaryl-coa reductase, IF—intermittent fasting, IR—insulin resistance, LPS—lipopolysaccharide, MC1568/TMP269/Panobinostat—selective HDAC inhibitors, miRNA—microRNA, mTOR—mechanistic target of rapamycin, NOD1—nucleotide-binding oligomerization domain-containing protein 1, OCN—osteocalcin, p53—tumor protein p53, PPARγ—peroxisome proliferator-activated receptor gamma, PTEN—phosphatase and tensin homolog, SAM—S-adenosylmethionine, SCFAs—short-chain fatty acids, SGBS—Simpson–Golabi–Behmel syndrome adipocyte cell line, SIRT—sirtuins, SVF—stromal vascular fraction, TGR5—G-protein-coupled bile acid receptor 1, TLR4/TRIF–MyD88—Toll-like receptor 4/TIR-domain-containing adapter-inducing interferon-β—myeloid differentiation primary response 88, ZF rats—Zucker fatty rats. ↑—increase in activity/level/expression; ↓—decrease in activity/level/expression.

**Table 4:** Principal epigenetic mechanisms influenced by bioactive dietary compounds, detailing the molecular pathways they target and the resulting metabolic effects associated with these modifications.

Epigenetic Mechanism	Enzyme/ Molecular Pathway	Bioactive Compound/Source	Epigenetic Biological Effect	Metabolic Effect	References
DNA methylation	DNMT1, DNMT3A/B (DNA methyltransferases)	EGCG (green tea), folates (leafy greens)	DNMT inhibition → demethylation of PGC1A and SIRT1 promoters	Increase glucose uptake, improved lipid metabolism	[20,141,294]
DNA methylation (methyl-donor pathway)	One-carbon metabolism (MTHFR, MAT, SAM/SAH cycle)	Folates, vitamin B_12_, betaine, choline	Increased methyl-group availability → stable DNA methylation	Regulation of genes involved in lipid and glucose metabolism	[123]
Histone acetylation/deacetylation	HAT, HDAC	Curcumin (turmeric), resveratrol (grapes), butyrate (SCFA)	HDAC inhibition → increased H3K9 and H4K12 acetylation → activation of antioxidant genes (*NRF2*, *PGC1A*)	Reduced oxidative stress and inflammation	[184,295,296]
Histone modifications (methylation/demethylation)	HMT (histone methyltransferases), HDM (histone demethylases)	Sulforaphane (broccoli), catechins	Altered H3K27 and H3K4 methylation → modulation of inflammatory gene expression (TNFα, IL-6)	Decreased inflammation and oxidative stress	[123,297]
microRNA regulation	miR-21, miR-122, miR-33, miR-34a	Polyphenols (green tea, berries), omega-3 fatty acids	Upregulation of anti-inflammatory microRNAs (e.g., miR-126); suppression of pro-inflammatory microRNAs	Lower CRP levels, improved lipid profile	[141,298]
Effects of SCFA (fiber fermentation)	HDAC, HAT	Butyrate, propionate (fiber fermentation products)	Butyrate as HDAC inhibitor → activation of genes regulating lipid and glucose metabolism	Increased insulin sensitivity, improved gut microbiota	[224,299]
Microbiome-epigenome interactions	SCFA, bacterial methyltransferases	Dietary fiber, probiotics, prebiotics	SCFA and microbial methyl-donor production → indirect modulation of host epigenome	Improved metabolic and immune profile	[300,301]

Abbreviations: DNMT—DNA methyltransferase, MTHFR—methylenetetrahydrofolate reductase, MAT—methionine adenosyltransferase, SAM/SAH—S-adenosylmethionine/S-adenosylhomocysteine, HAT—histone acetyltransferase, HDAC—histone deacetylase, HMT—histone methyltransferase, HDM—histone demethylase, SCFAs—short-chain fatty acids.

## Data Availability

No new data were created or analyzed in this study.
